# Autophagy-Related Chemoprotection against Sorafenib in Human Hepatocarcinoma: Role of FOXO3 Upregulation and Modulation by Regorafenib

**DOI:** 10.3390/ijms222111770

**Published:** 2021-10-29

**Authors:** Flavia Fondevila, Carolina Méndez-Blanco, Paula Fernández-Palanca, Tania Payo-Serafín, Jos van Pelt, Chris Verslype, Javier González-Gallego, José L. Mauriz

**Affiliations:** 1 Campus de Vegazana s/n, Institute of Biomedicine (IBIOMED), University of León, 24071 León, Spain; ffonp@unileon.es (F.F.); cmenb@unileon.es (C.M.-B.); pferp@unileon.es (P.F.-P.); tpayos00@estudiantes.unileon.es (T.P.-S.); jgonga@unileon.es (J.G.-G.); 2 Centro de Investigación Biomédica en Red de Enfermedades Hepáticas y Digestivas (CIBERehd), Instituto de Salud Carlos III, Av. de Monforte de Lemos 5, 28029 Madrid, Spain; 3 Laboratory of Clinical Digestive Oncology, Department of Oncology, Leuven Cancer Institute (LKI), KU Leuven and University Hospitals Leuven, 3000 Leuven, Belgium; jos.vanpelt@kuleuven.be (J.v.P.); chris.verslype@uzleuven.be (C.V.)

**Keywords:** autophagy, FOXO3, hepatocarcinoma, regorafenib, resistance, sorafenib

## Abstract

Early acquisition of sorafenib resistance is responsible for the dismal prognosis of advanced hepatocarcinoma (HCC). Autophagy, a catabolic process involved in liver homeostasis, has been associated with chemosensitivity modulation. Forkhead box O3 (FOXO3) is a transcription factor linked to HCC pathogenesis whose role on autophagy-related sorafenib resistance remains controversial. Here, we unraveled the linkage between autophagy and sorafenib resistance in HCC, focusing on the implication of FOXO3 and its potential modulation by regorafenib. We worked with two HepG2-derived sorafenib-resistant HCC in vitro models (HepG2S1 and HepG2S3) and checked HCC patient data from the UALCAN database. Resistant cells displayed an enhanced basal autophagic flux compared to HepG2, showing higher autophagolysosome content and autophagy markers levels. Pharmacological inhibition of autophagy boosted HepG2S1 and HepG2S3 apoptosis and subG1 cells, but reduced viability, indicating the cytoprotective role of autophagy. HCC samples displayed higher FOXO3 levels, being associated with shorter survival and autophagic genes expression. Consistently, chemoresistant in vitro models showed significant FOXO3 upregulation. FOXO3 knockdown suppressed autophagy and caused resistant cell death, demonstrating that overactivation of such pro-survival autophagy during sorafenib resistance is FOXO3-dependent; a cytoprotective mechanism that the second-line drug regorafenib successfully abolished. Therefore, targeting FOXO3-mediated autophagy could significantly improve the clinical efficacy of sorafenib.

## 1. Introduction

Hepatocarcinoma (HCC), one of the deadliest tumors worldwide [1], is usually diagnosed at advanced stages [2]. The multi-target tyrosine kinase inhibitor (TKI) sorafenib, the first drug approved for advanced HCC, still constitutes the standard first-line treatment for patients with advanced disease [3]. However, patients actually obtain a short survival benefit due to the apparition of sorafenib-resistant tumor hepatocytes within the first six months of treatment [2,3,4]. Therefore, how to prevent or overcome early acquisition of sorafenib resistance has become an imperative challenge in the HCC landscape.

Apart from tumor genetic heterogeneity [5], hypoxic microenvironment [6,7], alteration of key signaling pathways or autophagy have been suggested to be involved in more aggressive tumor behavior and the loss of sensitivity to sorafenib [5]. Autophagy is an evolutionarily conserved catabolic and recycling process by which damaged or redundant cellular components are engulfed into double-membrane vesicles—autophagosomes—in order to be degraded through the lysosomal pathway [8,9,10]. This process comprises autophagosome formation and maturation, autophagolysosome formation by fusion with the lysosome, and final cargo degradation [8,10]. This self-digestive mechanism plays an important physiological role in the liver as it is the major metabolic organ [9], and dysregulation of autophagy has been even related to several hepatic diseases including HCC [11].

It is known that autophagy plays a double function in HCC depending on the specific cellular context, protecting against HCC initiation and progression, but also promoting HCC malignancy when the tumor is already established [9]. Regarding sorafenib resistance, diverse investigations reported opposing effects of autophagy under sorafenib treatment, finding that autophagy can encourage sorafenib resistance or efficacy in HCC [3]. This fact illustrates the complexity of autophagy, a dual role process involved in sorafenib failure, that needs to be fully understood at a molecular level in order to design autophagy-targeting therapies able to improve advanced HCC clinical settings.

Forkhead box O3 (FOXO3), an important member of the FOXO subfamily of transcription factors, has shown to transcriptionally upregulate several autophagy-related markers such as unc-51-like autophagy activating kinase 1 (ULK1), Beclin-1 and microtubule associated protein 1 light chain 3 (LC3) [12], controlling autophagy even in cancer cells [13]. Contrary roles of FOXO3 have been found in different cancer types, acting as a tumor suppressor but also favoring tumor progression under certain conditions [14,15,16]. Moreover, it has been suggested that FOXO3 could participate in the autophagy-mediated modulation of sensitivity to chemotherapy in tumor cells [17,18,19,20].

There is an urgent need to identify new targetable molecular mechanisms for improving advanced HCC prognosis. However, despite the potential connection between FOXO3-related autophagy and sorafenib resistance, this interesting relationship has been barely studied, and only two articles have analyzed the topic [21,22]. This previous research was mostly conducted under specific hypoxic conditions and, surprisingly, disclosures regarding how FOXO3 modulates autophagy and sorafenib sensitivity in HCC are opposite [21,22]. While Liang et al. [22] reported that hypoxia activates FOXO3, leading to autophagy induction and sorafenib resistance, Lin et al. [21] contrarily suggested that FOXO3 overexpression under hypoxia could reverse sorafenib resistance by autophagy inhibition. Additionally, several experimental inaccuracies, such as the employment of non-resistant HCC models [22], or the exclusive usage of the unspecific autophagy inhibitor 3-methyladenine to monitor autophagic flux [21,22], are found in these previous studies.

Thus, the exact crosstalk between FOXO3, autophagy, and sorafenib resistance in HCC remains fully unclear and needs to be further and consistently studied, especially using adequate sorafenib-resistant HCC in vitro models and exploring tumor cell response under normal oxygen conditions. In order to achieve reliable and confident results, in vitro experiments were carried out in the present research using two well-established sorafenib-resistant HCC models that were independently generated from HepG2 cell line, thereby employing adequate cell types that resemble, in a more precise way, in vivo and human chemoresistant conditions. The above-mentioned studies [21,22] mostly evaluated sorafenib sensitivity under specific hypoxic conditions. However, we fully examined the role played by FOXO3 on autophagy-mediated sorafenib resistance under normoxia, thereby covering all oxygen-related microenvironmental conditions and unraveling sorafenib-resistant HCC cell response under oxygen availability. Besides, we independently used two specific inhibitors of late autophagy (bafilomycin A1 and chloroquine) to assess autophagic flux and the impact of autophagy blockage.

Apart from working with suitable in vitro models, we also analyzed HCC sample data with the aim of determining the role played by FOXO3 in HCC patients and its potential linkage with autophagy before conducting in vitro experiments. For this purpose, we used the UALCAN database, an informative and innovative tool that has never been used in previous related papers to analyze HCC patient data. Therefore, prior to exploring the involvement of FOXO3 in autophagy-mediated sorafenib resistance in vitro, we firmly set the basis regarding FOXO3 expression in HCC specimens, verified the association with survival-related prognosis, and ensured the correlation of FOXO3 with the expression of crucial autophagy genes in HCC patients.

As a novelty, we also employed the main second-line drug approved for sorafenib-refractory HCC patients—regorafenib—to confirm our FOXO3 and autophagy-related findings in sorafenib-resistant HCC. Thus, we demonstrated for the first time the modulatory effects of the TKI regorafenib on FOXO3 and autophagy in sorafenib-resistant HCC.

Our study successfully confirmed that aberrant FOXO3 upregulation is linked to worse HCC phenotypes, being responsible for the induction of a pro-survival autophagy that contributes to sorafenib resistance acquisition in HCC. Additionally, we proved for the first time that regorafenib exerts its anti-tumor actions in chemoresistant HCC by impairing such cytoprotective FOXO3 upregulation and autophagy induction. Thus, targeting FOXO3-induced autophagy emerges as a novel and suitable therapeutic approach to improve sorafenib-based treatment in late-stage HCC.

## 2. Results

### 2.1. Sorafenib-Resistant HCC In Vitro Models Display an Enhanced Basal Autophagic Flux

Early acquisition of sorafenib resistance has become a frequent issue in HCC that needs to be urgently solved [2,4,5]. Autophagy has been included among the mechanisms potentially involved in the refractoriness to sorafenib of HCC cells [3,5,8], but its modulation by sorafenib is complex, depends on the cellular context, and remains not fully understood [2,3,5,8,23]. In this study, we contributed to clarifying this knowledge by investigating the implication of autophagy in the acquisition of sorafenib resistance in HCC, focusing on exactly determining the controversial FOXO3-related molecular basis underlying this mechanism.

**Figure 1:**
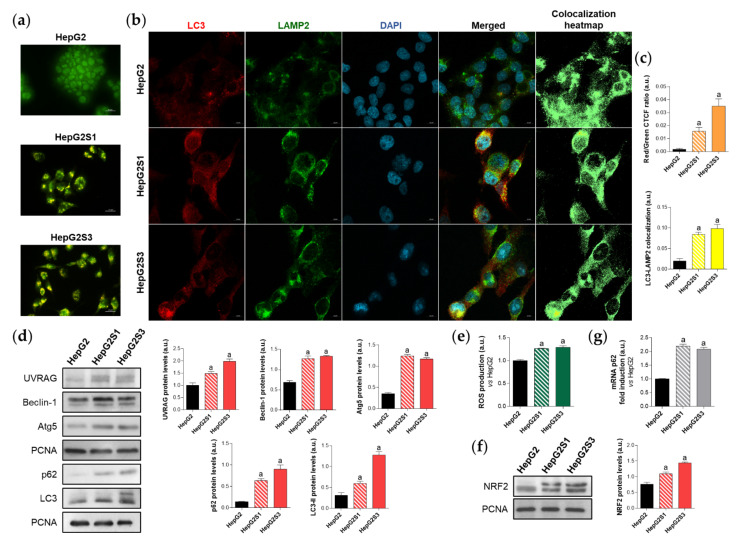
Comparison of basal autophagic status between HepG2S1 and HepG2S3 sorafenib-resistant lines and sorafenib-sensitive HepG2 parental cells. (**a**) Fluorescence microscopy images from acridine orange staining. Magnification 40×, scale bar 25 µm; (**b**) Confocal microscopy images from microtubule-associated protein 1 light chain 3 (LC3)-lysosomal associated membrane protein 2 (LAMP2) immunofluorescence. Red fluorescence, LC3. Green, LAMP2. Blue, cell nucleus. Yellow fluorescence in merged channel indicates LC3-LAMP2 colocalization. Magnification 63×, scale bar 10 µm. Corresponding LC3-LAMP2 colocalization heatmaps obtained using ImageJ are also shown; (**c**) Quantification of red/green corrected total cell fluorescence (CTCF) ratio from (**a**) and LC3-LAMP2 colocalization from (**b**), upper and lower bar graphs, respectively; (**d**) Representative immunoblots showing protein expression of several autophagy markers. Densitometry reading of each band (relative to the corresponding original proliferating cell nuclear antigen (PCNA) band) is shown; (**e**) Measurement of reactive oxygen species (ROS) production; (**f**) Immunoblots showing nuclear factor erythroid 2-like 2 (NRF2) protein levels. Densitometry reading of each band is shown; (**g**) Assessment of sequestosome 1 (p62) mRNA levels. Data from (**c**–**g**) are expressed as mean values of arbitrary units (a.u.) ± SD of three independent experiments. ^a^ *p* < 0.05 vs. HepG2 cells.

First, in order to evaluate the effect of prolonged sorafenib treatment on autophagy modulation in our HCC in vitro models, we started with the assessment of the basal autophagic status of the two sorafenib-resistant lines and the parental cells HepG2. Results from acridine orange staining plus fluorescence microscopy showed a prominent increase of autophagolysosome amount (orange-yellow fluorescence) in both sorafenib-resistant lines, especially in HepG2S3 (Figure 1a,c). However, autophagy seemed not to be induced in the sorafenib-sensitive HepG2 cells as orange-yellow puncta indicating autophagolysosomes were almost not detected (Figure 1a,c). Confocal microscopy images (merged channel) and colocalization heatmaps from LC3 and lysosomal associated membrane protein 2 (LAMP2) immunofluorescence, an autophagosome and lysosome marker respectively, denoted an enhanced overlap of red (LC3) and green (LAMP2) fluorescence in both resistant cell lines (Figure 1b,c); which indicated positive colocalization between both organelles and autophagy induction.

Additionally, protein expression of the autophagy markers UV radiation resistance-associated (UVRAG), Beclin-1, autophagy-related 5 (Atg5), sequestosome 1 (p62) and LC3 was compared between HepG2S1 and HepG2S3 and the parental line. In a previous work of our research group, we noticed the impossibility of using any cytoskeletal protein such as β-actin as a loading control in Western blot assays because of its expression instability between our HCC in vitro models. Thus, proliferating cell nuclear antigen (PCNA), whose validity has been confirmed in different species and in our HCC cell lines [6], was chosen as the housekeeping protein to accomplish this research. Protein levels of UVRAG, Beclin-1 and Atg5 were significantly upregulated in the sorafenib-resistant cells compared to HepG2 (Figure 1d). Furthermore, HepG2S1 and particularly HepG2S3 showed higher protein levels of both p62 and LC3-II (the lipidated form of LC3) (Figure 1d). However, autophagy induction is traditionally characterized by increased LC3-II levels accompanied by decreased expression of p62, which is a substrate of autophagic degradation [24,25]. Therefore, all results from HepG2S1 and HepG2S3 resistant lines, with the exception of p62 expression, most likely suggest that autophagy was induced in HCC cells upon sustained sorafenib treatment.

It is known that p62 is transcriptionally upregulated by the nuclear factor erythroid 2-like 2 (NRF2), a transcription factor whose stabilization is prompted by chemotherapy-induced oxidative stress [26,27]. In order to find an explanation for the augmented p62 expression in our sorafenib-resistant cells, we analyzed the NRF2/p62 axis by measuring reactive oxygen species (ROS) production, NRF2 expression and p62 transcription levels in parallel. As expected, both HepG2S1 and HepG2S3 resistant lines exhibited higher ROS, NRF2, and p62 mRNA levels than HepG2 cells (~2-fold mRNA levels vs. HepG2) (Figure 1e–g). These findings support the involvement of this oxidative stress-dependent mechanism in the upregulation of p62 in our sorafenib-resistant cells, where p62 de novo synthesis seems to overcome its autophagic degradation.

### 2.2. Inhibition of Autophagy Leads to Sorafenib-Resistant Cell Death

**Figure 2:**
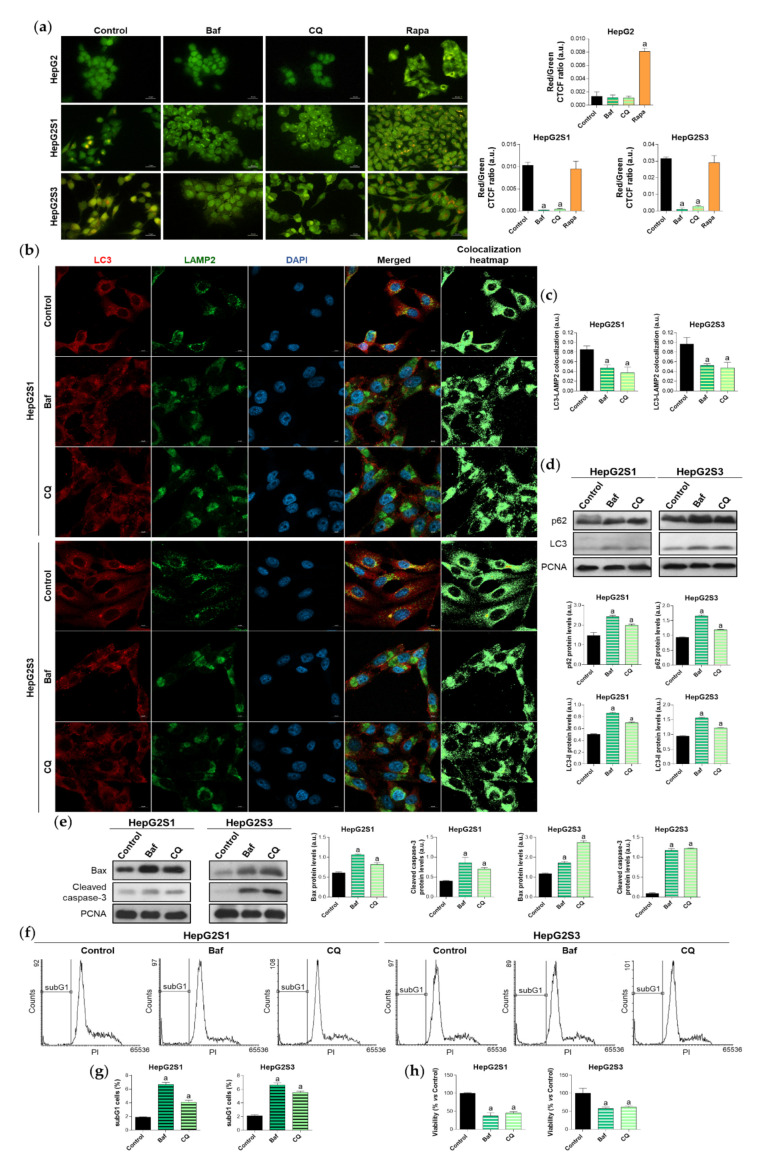
Effect of autophagic flux modulation on basal autophagy, cell death, and viability. Hepatocarcinoma (HCC) cell lines were treated with 100 nM bafilomycin A1 (Baf), 40 µM chloroquine (CQ) or 200 nM rapamycin (Rapa) for 24 h. (**a**) Fluorescence microscopy images from acridine orange staining. Magnification 40×, scale bar 25 µm. Bar graphs representing the quantification of red/green CTCF ratio are also shown; (**b**) Confocal microscopy images from LC3-LAMP2 immunofluorescence. Red fluorescence, LC3. Green, LAMP2. Blue, cell nucleus. Yellow fluorescence in merged channel indicates LC3-LAMP2 colocalization. Magnification 63×, scale bar 10 µm. Corresponding LC3-LAMP2 colocalization heatmaps obtained using ImageJ are also shown; (**c**) Bar graphs representing the quantification of LC3-LAMP2 colocalization from (**b**); (**d**) Immunoblots showing p62 and LC3 turnover. Densitometry reading of each band is shown; (**e**) Immunoblots showing protein expression of Bcl-2 associated X apoptosis regulator (Bax) and cleaved caspase-3. Densitometry reading of each band is shown; (**f**,**g**) Analysis of subG1 cell population and its quantification; (**h**) Evaluation of cell viability. Data from (**a**), (**c**–**e**) are expressed as mean values of arbitrary units (a.u.) ± SD of three independent experiments. Data from (**g**,**h**) are expressed as the percentage of mean values ± SD of three independent experiments. ^a^ *p* < 0.05 vs. control (non-treated) cells.

To confirm the induction of autophagy in our sorafenib-resistant cells, we evaluated the impact of the individual treatment with different compounds that modulate autophagy flux on the autophagic basal status. Specifically, cells were exposed for 24 h to bafilomycin A1 and chloroquine, two recognized inhibitors of autophagosome-lysosome fusion, or rapamycin, one of the most common autophagy inducers [24]. Basal autophagy of HepG2S1 and HepG2S3 lines was only disturbed by adding bafilomycin A1 or chloroquine, which caused a marked reduction in autophagolysosome cellular content that was similar for both resistant cell lines (Figure 2a). Contrariwise, autophagy inhibition did not report any changes in HepG2 cells, while rapamycin effectively induced autophagolysosome formation (Figure 2a). Moreover, blocking the fusion of autophagosomes with lysosomes resulted in the diminution of LC3-LAMP2 colocalization in the resistant lines (~0.5-fold vs. non-treated cells) (Figure 2b,c), which agrees with the results derived from acridine orange staining.

Single treatment of HepG2S1 and HepG2S3 cells with the inhibitors of autophagic flux further enhanced p62 and LC3-II levels with respect to its corresponding steady-state expression (Figure 2d), indicating autophagic degradation blockage and autophagosomes accumulation. In this experiment, bafilomycin A1 was more effective inducing p62 and LC3-II accumulation than chloroquine (Figure 2d), while both agents were able to similarly reduce autophagolysosome number in HepG2S1 and HepG2S3 cells (Figure 2a–c). Altogether, these results confirm that our sorafenib-resistant lines are autophagy-competent and that HCC cells induced autophagy in response to long-term sorafenib administration.

Due to the importance of establishing the effect of the sorafenib-mediated activation of autophagy on cell survival, we next analyzed the role played by this double-function process in the resistant lines. We used bafilomycin A1 or chloroquine for 24 h to impair autophagic flux and, afterwards, the expression of the apoptotic markers Bcl-2 associated X apoptosis regulator (Bax) and cleaved caspase-3, subG1 cell population, and cell viability were evaluated. Autophagy abolition by these two agents significantly elevated protein expression of the above-mentioned apoptotic markers in both resistant cell lines (Figure 2e). Besides, analysis of cell cycle by flow cytometry revealed a notable augment in the percentage of HepG2S1 and HepG2S3 subG1 cells when autophagy flux was suppressed; triplicating subG1 population when bafilomycin A1 was added, and approximately doubling it when resistant cells were subjected to chloroquine (Figure 2f,g). Accordingly, cell viability showed a 50% reduction when autophagy was impaired (Figure 2h). Taken together, these findings suggest that our chemoresistant HCC cell lines were able to activate a cytoprotective autophagy to surpass the anti-tumor actions of sorafenib during the process of resistance acquisition.

### 2.3. FOXO3 Is Upregulated in HCC Samples and Sorafenib-Resistant Lines, Being also Correlated with Autophagy-Related Genes Expression and Linked to Poor Patient Survival

The underlying molecular mechanisms of autophagy-related sorafenib resistance are still unclear, which precludes the autophagy-mediated sorafenib resistance fading and compromises advanced HCC patient outcomes. Meanwhile, it has been suggested that deregulation of FOXO3 expression or activity could provoke tumor development and boost the apparition of more aggressive tumor phenotypes such as those with chemoresistance [13,21,22,28].

**Figure 3:**
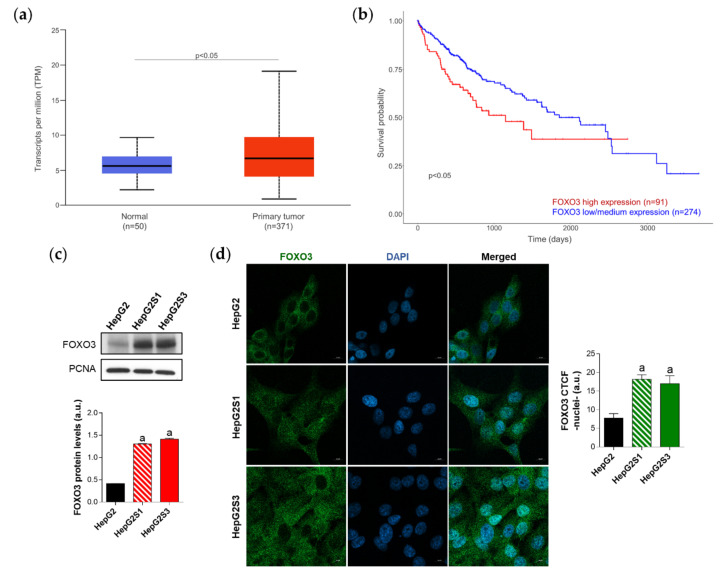
Characterization of forkhead box O3 (FOXO3) expression in HCC patient samples and sorafenib-resistant HCC in vitro models. (**a**) Comparison of FOXO3 expression between HCC and normal liver tissues. *p* < 0.05 significant differences between groups; (**b**) Impact of FOXO3 expression on HCC patient survival rate. *p* < 0.05 significant differences between high (red) and low/medium (blue) FOXO3 levels; (**c**) Immunoblots from the in vitro analysis of FOXO3 expression. Densitometry reading of each band is shown; (**d**) Evaluation of FOXO3 nuclear translocation employing confocal microscopy and FOXO3 immunofluorescence. Green fluorescence, FOXO3. Blue, cell nucleus. Magnification 63×, scale bar 10 µm. Bar graph from the quantification of nuclear green fluorescence (FOXO3) is also shown. Data from (**c**,**d**) are expressed as mean values of arbitrary units (a.u.) ± SD of three independent experiments. ^a^ *p* < 0.05 vs. HepG2 cells.

Thus, FOXO3 could constitute a central hub connecting HCC sorafenib resistance and the overactivation of pro-survival autophagy. To address this hypothesis, we first checked the expression of this transcription factor in human HCC samples and normal liver tissues, as well as the influence of FOXO3 levels on HCC patient survival. For this purpose, we employed the TCGA gene analysis tool from the UALCAN database. Based on the results from 50 healthy liver samples and 371 HCC patients, FOXO3 is significantly greater expressed in tumor samples (Figure 3a). Likewise, overall survival analysis of 365 HCC patients (91 with low/medium and 274 with high FOXO3 levels) indicated that individuals overexpressing this factor have significantly lower survival rates (Figure 3b). Therefore, FOXO3 appears to be elevated upon HCC development, being associated with a poor prognosis.

Diverse articles defend that FOXO3 is able to transcriptionally upregulate several autophagy markers, which can lead to autophagy activation [12]. This is the reason why we also investigated the autophagy-related genes whose expression correlates with FOXO3 in HCC samples. Transcriptional targets of FOXO3 such as PIK3C3, ULK1, ATG12, BECN1, and ATG4B predominantly showed a moderate degree of positive correlation with FOXO3; reaching a Pearson-correlation coefficient (Pearson-CC) of +0.51 in the case of PIK3C3, which was the most strongly correlated target (Table 1) (Figure S1). In addition, expression of multiple other genes found in the autophagy KEGG pathway (hsa04140) were shown to be positively correlated with FOXO3 in HCC patients, highlighting the modest correlation observed by ATG5 (+0.62), UVRAG (+0.55), PIK3R4 (+0.51), ATG16L1 (+0.48), AMBRA1 (+0.47), ATG3 (+0.35), and RB1CC1 (+0.34) (Table 1) (Figure S1); altogether supporting an enhanced autophagic status. It should be mentioned that all the observed correlations were positive and significant (*p* < 0.0001), not finding autophagic genes negatively correlated with FOXO3 in HCC samples. These results suggest that high FOXO3 expression is not only associated with disappointing patient outcomes, but also with a pro-autophagic environment in HCC.

Since high levels of FOXO3 in HCC patients seemed to be linked to a worse prognosis, we assessed FOXO3 expression and subcellular location in vitro to evaluate the possible FOXO3 upregulation in our sorafenib-resistant lines, which both resemble a more advanced disease stage. Moreover, it is worth mentioning that excessive oxidative stress is able to stimulate the transcriptional activity of FOXO3 [27] and, in this study, sorafenib-resistant cell lines had shown enhanced ROS levels with respect to parental HepG2 (Figure 1e). Western blot results revealed a pronounced increase of FOXO3 expression in HepG2S1 and HepG2S3 lines in comparison to sorafenib-sensitive parental cells (Figure 3c). Besides, quantification of confocal microscopy images for FOXO3 immunofluorescence detected a significant increment in nuclear retention of FOXO3 in sorafenib-resistant cells (Figure 3d). These results indicate that long-term sorafenib treatment generates an oxidative stress atmosphere that could lead to FOXO3 overexpression and nuclear translocation, where this factor exerts its transcriptional activity.

### 2.4. FOXO3 Mediates the Overactivation of Autophagy in Sorafenib-Resistant HCC Cells

There is evidence supporting that FOXO3 deregulation is associated with autophagy modulation, being related to the loss or gain of drug sensitivity in different cancer types [17,18,19,20,29]. However, the linkage between sorafenib response, autophagy, and FOXO3 in HCC remains largely unknown.

**Figure 4:**
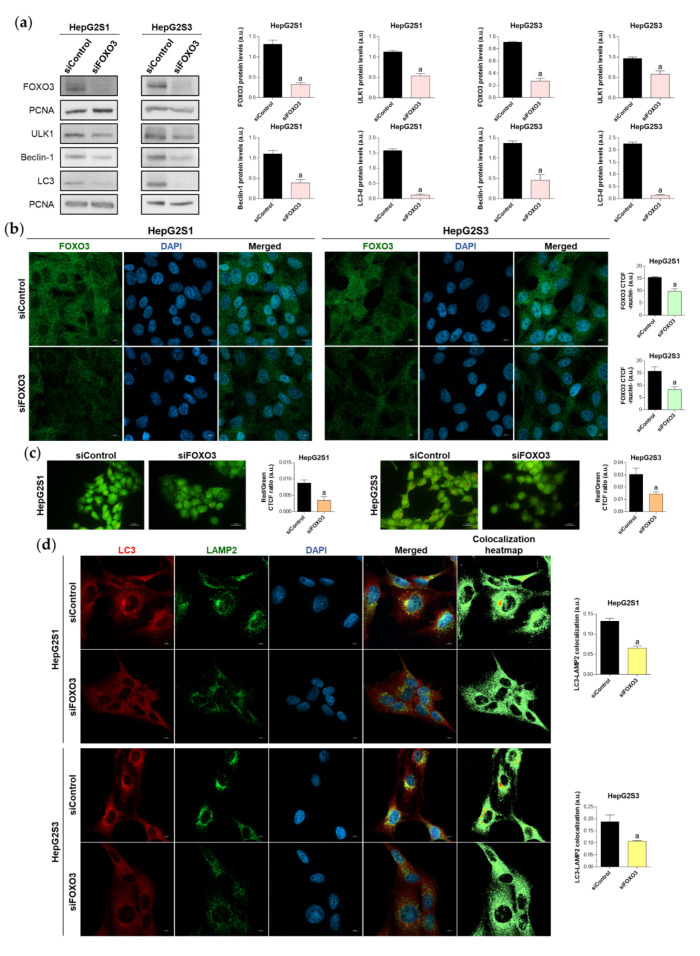
Effect of FOXO3 knockdown on the basal autophagic status of HepG2S1 and HepG2S3 sorafenib-resistant cell lines. All the assays were carried out at 48 h post-silencing. (**a**) Immunoblots of FOXO3 and several autophagy targets. Densitometry reading of each band is shown; (**b**) Analysis of FOXO3 nuclear translocation by confocal microscopy and FOXO3 immunofluorescence. Green fluorescence, FOXO3. Blue, cell nucleus. Magnification 63×, scale bar 10 µm. Quantification of nuclear green fluorescence (FOXO3) is also shown; (**c**) Fluorescence microscopy images from acridine orange staining. Magnification 40×, scale bar 25 µm. Bar graphs representing the quantification of red/green CTCF ratio are also shown; (**d**) Confocal microscopy images from LC3-LAMP2 immunofluorescence. Red fluorescence, LC3. Green, LAMP2. Blue, cell nucleus. Yellow fluorescence in merged channel indicates LC3-LAMP2 colocalization. Magnification 63×, scale bar 10 µm. Corresponding LC3-LAMP2 colocalization heatmaps obtained using ImageJ and bar graphs representing the quantification of LC3-LAMP2 colocalization are also shown. Data from (**a**–**d**) are expressed as mean values of arbitrary units (a.u.) ± SD of three independent experiments. ^a^ *p* < 0.05 vs. siControl cells.

Given that patient data suggested a connection between FOXO3 and autophagy induction in HCC, and that our sorafenib-resistant models displayed FOXO3 upregulation, we then analyzed the direct effect of FOXO3 knockdown by siRNA transfection on the enhanced basal autophagy of HepG2S1 and HepG2S3. FOXO3 was efficiently silenced at 48 h post-transfection, achieving around 70% FOXO3 knockdown for both sorafenib-resistant cell lines (Figure 4a). In agreement with this result, nuclear retention of this factor was notably lower in HepG2S1 and HepG2S3 FOXO3-silenced cells (Figure 4b). Consequently, expression of ULK1, Beclin-1, and LC3, three autophagy markers that FOXO3 controls at the transcriptional level, was significantly reduced after FOXO3 silencing in both sorafenib-resistant lines (Figure 4a). In comparison to non-silenced resistant cells, protein levels of ULK1 and Beclin-1 approximately declined by half, whereas LC3-II expression was dramatically decreased by more than 90% (Figure 4a). Furthermore, FOXO3 knockdown abolished the autophagic flux induced by sorafenib in HepG2S1 and HepG2S3 cells, finding an evident decrease in autophagolysosome content and LC3-LAMP2 colocalization (Figure 4c,d). Altogether, these findings indicate that the induction of autophagy after sorafenib sustained treatment is mediated, at least in part, by FOXO3 upregulation.

### 2.5. Survival of Sorafenib-Resistant Cell Lines Is FOXO3-Dependent

To verify the idea that FOXO3-induced autophagy could be essential for sorafenib-resistant cell survival, we tested the impact of FOXO3 knockdown on HepG2S1 and HepG2S3 cell proliferation, viability, and apoptosis at 48 h post-transfection.

**Figure 5:**
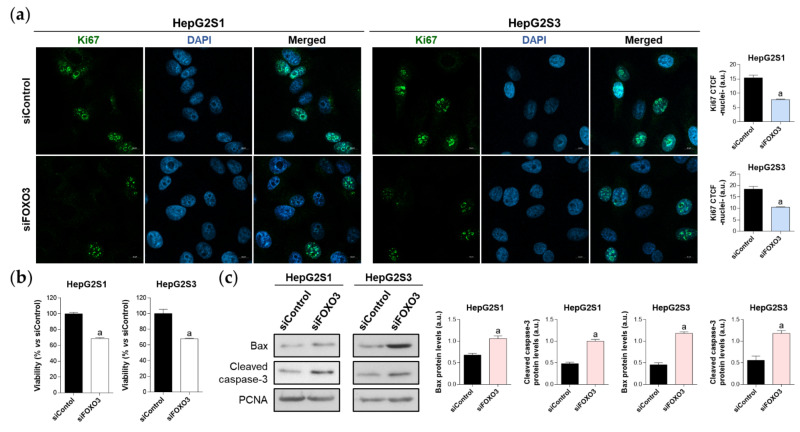
Effect of FOXO3 knockdown on HepG2S1 and HepG2S3 cell proliferation, viability, and apoptosis. All the assays were carried out at 48 h post-silencing. (**a**) Assessment of cell proliferation using Ki67 immunofluorescence and confocal microscopy. Green fluorescence, Ki67. Blue, cell nucleus. Magnification 63×, scale bar 10 µm. Quantification of nuclear green fluorescence (Ki67) is also shown; (**b**) Determination of cell viability; (**c**) Immunoblots showing Bax and cleaved caspase-3 protein levels. Densitometry reading of each band is shown. Data from (**a**,**c**) are expressed as mean values of arbitrary units (a.u.) ± SD of three independent experiments. Data from (**b**) are expressed as the percentage of mean values ± SD of three independent experiments. ^a^ *p* < 0.05 vs. siControl cells.

The proliferation rate was evaluated using Ki67 immunofluorescence and confocal microscopy. FOXO3-silenced resistant cells showed a reduced proliferation index, which was decreased by nearly 50% compared to control cells (Figure 5a). Similarly, analysis of cell viability employing the luminescent assay CellTiter-Glo^®^ enoted a significant ~30% viability reduction in both sorafenib-resistant lines (Figure 5b). Moreover, FOXO3 knockdown yielded apoptosis enhancement as shown by higher protein levels of Bax and cleaved caspase-3 in siFOXO3 HepG2S1 and HepG2S3 cells (Figure 5c). Hence, these findings indicate that FOXO3 upregulation during the development of sorafenib resistance in HCC protects chemoresistant hepatocytes from the anti-tumor actions of sorafenib.

### 2.6. Regorafenib Impairs FOXO3-Mediated Autophagy in Sorafenib-Resistant HCC Lines

Systemic therapy with regorafenib constitutes the recommended second-line treatment when sorafenib sensitivity is lost [30]. Among the anti-tumor effects of regorafenib, this TKI could modulate the autophagy pathway to counteract tumor cell survival [31], but the effect of regorafenib on FOXO3 and autophagy regulation in sorafenib-resistant HCC has not been studied yet.

**Figure 6:**
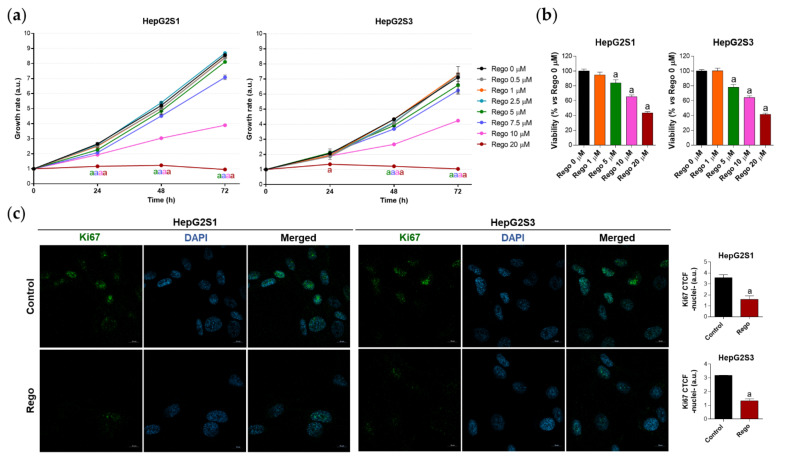
Evaluation of HepG2S1 and HepG2S3 cell growth, viability, and proliferation after treatment with regorafenib. (**a**) Determination of cell growth during 24, 48 and 72 h under several concentrations of regorafenib (Rego) ranging from 0 to 20 µM using crystal violet staining. ^a^ *p* < 0.05 significant differences between each dosage and Rego 0 µM at each timepoint; (**b**) Analysis of cell viability at 48 h post-treatment with diverse concentrations of regorafenib ranging from 0 to 20 µM using 3-(4,5-dimethylthiazol-2-yl)-2,5-diphenyl-tetrazolium bromide (MTT) assay. ^a^ *p* < 0.05 vs. Rego 0 µM; (**c**) Assessment of proliferation rate by Ki67 immunofluorescence and confocal microscopy after treatment with 20 µM regorafenib for 48 h. Green fluorescence, Ki67. Blue, cell nucleus. Magnification 63×, scale bar 10 µm. Quantification of nuclear green fluorescence (Ki67) is also shown. ^a^ *p* < 0.05 vs. control (non-treated) cells. Data from (**a**,**c**) are expressed as mean values of arbitrary units (a.u.) ± SD of three independent experiments. Data from (**b**) are expressed as the percentage of the mean values ± SD of three independent experiments.

Therefore, we decided to treat our chemoresistant HCC cells with this second-line drug to test whether the efficacy of regorafenib under a cellular context of sorafenib resistance is partially due to the inhibition of the FOXO3-mediated autophagy. This would definitely confirm the involvement of such FOXO3-dependent autophagy in the refractoriness to sorafenib of resistant HCC cells, also verifying for the first time the beneficial modulatory action of regorafenib on this chemoresistant mechanism. In order to assess regorafenib efficacy in our sorafenib-resistant cells, we first analyzed the impact on cell growth and viability of a subset of regorafenib concentrations. Results displayed in Figure 6a show the inhibition of cellular growth caused by regorafenib along 24, 48, and 72 h, an effect that was concentration- and time-dependent. At 48 h post-treatment, only 5, 7.5, 10 and 20 µM regorafenib significantly decreased HepG2S1 and HepG2S3 growth in comparison to non-treated cells (Figure 6a). Similarly, the viability of sorafenib-resistant lines after 48 h with regorafenib was declined in a dose-dependent way (Figure 6b). Ki67-based proliferation index was reduced by half in both HepG2S1 and HepG2S3 after 48 h-exposition to 20 µM regorafenib (Figure 6c), the dose that was established as ~IC_50_ for our resistant cells considering the results from viability assays.

**Figure 7:**
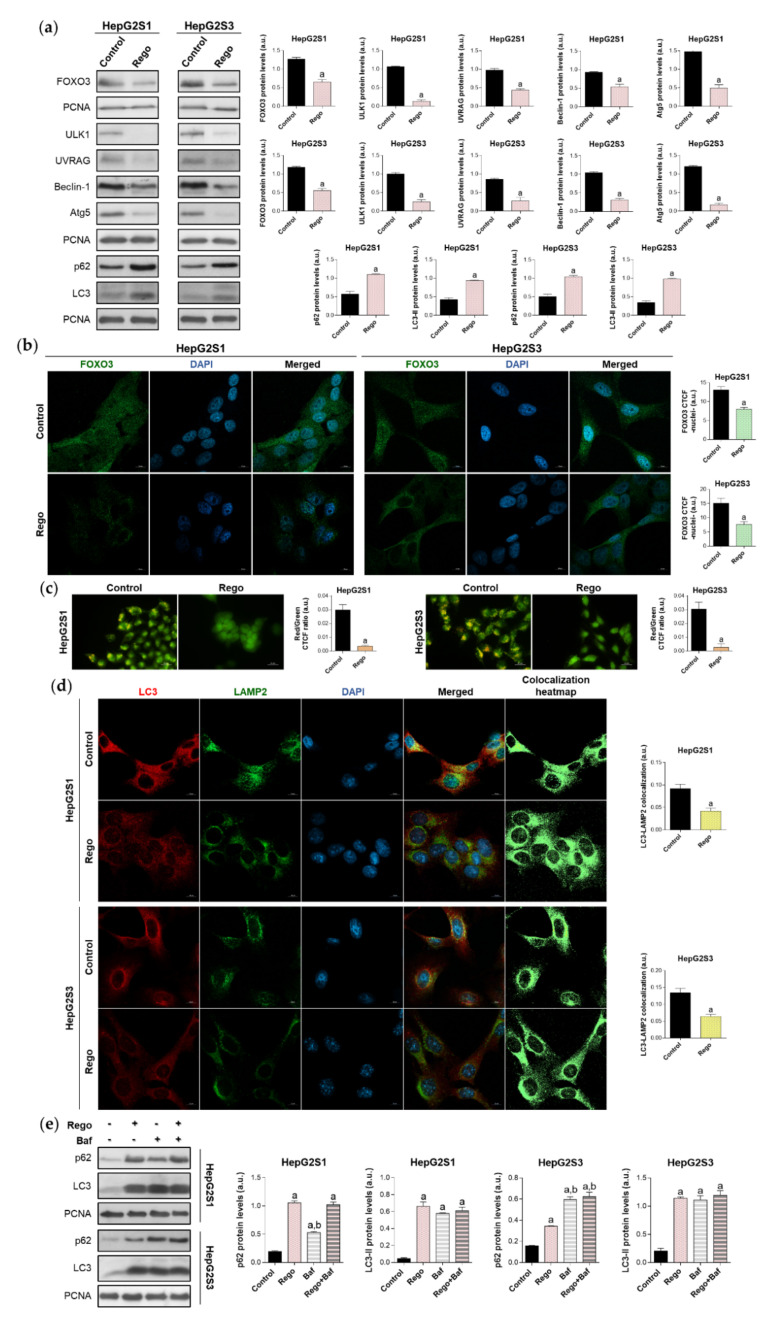
Impact of regorafenib treatment on the induced autophagy of HepG2S1 and HepG2S3 sorafenib-resistant cell lines. HCC cells were treated with 20 µM regorafenib (Rego) for 48 h. (**a**) Immunoblots of FOXO3 and autophagy-related markers. Densitometry reading of each band is shown; (**b**) Assessment of FOXO3 nuclear translocation by confocal microscopy and FOXO3 immunofluorescence. Green fluorescence, FOXO3. Blue, cell nucleus. Magnification 63×, scale bar 10 µm. Quantification of nuclear green fluorescence (FOXO3) is also shown; (**c**) Fluorescence microscopy images from acridine orange staining. Magnification 40×, scale bar 25 µm. Bar graphs representing the quantification of red/green CTCF ratio are also shown; (**d**) Confocal microscopy images from LC3-LAMP2 immunofluorescence. Red fluorescence, LC3. Green, LAMP2. Blue, cell nucleus. Yellow fluorescence in merged channel shows LC3-LAMP2 colocalization. Magnification 63×, scale bar 10 µm. Corresponding LC3-LAMP2 colocalization heatmaps obtained using ImageJ and bar graphs representing the quantification of LC3-LAMP2 colocalization are also shown; (**e**) Immunoblots showing p62 and LC3 turnover after single or combined treatment with regorafenib and 100 nM bafilomycin A1 (Baf). In the case of bafilomycin A1 single administration, cells were exposed to this agent for 24 h while, for the co-treatment, cells were subjected to regorafenib for 48 h in presence of bafilomycin A1 during the last 24 h. Densitometry reading of each band is shown. Data from (**a**–**e**) are expressed as mean values of arbitrary units (a.u.) ± SD of three independent experiments. ^a^ *p* < 0.05 vs. control (non-treated) cells, ^b^ *p* < 0.05 vs. regorafenib-treated cells.

We subsequently assessed FOXO3 expression and subcellular location, as well as global autophagic status, after treatment with 20 µM regorafenib. 48 h exposure to this drug significantly downregulated FOXO3, ULK1, UVRAG, Beclin-1, and Atg5 levels, decreasing the protein expression by more than 50% in almost all cited markers (Figure 7a). Moreover, nuclear translocation of FOXO3 was considerably inhibited by regorafenib (Figure 7b), which also substantially diminished autophagolysosome content (Figure 7c) and, therefore, colocalization between both organelles (Figure 7d). Otherwise, the addition of regorafenib to sorafenib-resistant cells for 48 h entailed greater p62 and LC3-II protein expression in both cell lines (Figure 7a). Therefore, we addressed the possibility that this drug could also inhibit autophagy at later phases. This would explain the general suppression of FOXO3-mediated autophagy in the presence of both high LC3-II and p62 levels.

To evaluate this hypothesis, we compared the effect of single and combined treatment with bafilomycin A1 and regorafenib on LC3-II and p62 turnover in HepG2S1 and HepG2S3 cells. Regorafenib or bafilomycin A1 alone led to a similar LC3-II accumulation; while co-treatment increased steady-state levels, but no differences were detected compared to single treatments (Figure 7e). Bafilomycin A1 elevated p62 expression, being this increment lower and higher than that occasioned by regorafenib in HepG2S1 and HepG2S3, respectively (Figure 7e). However, coadministration reached the same p62 levels as regorafenib in HepG2S1 cells, and bafilomycin A1 in HepG2S3 (Figure 7e). Overall, combined treatment did not further accumulate LC3-II or p62 (Figure 7e). Global interpretation of these results suggests that regorafenib also blocks autophagosome-lysosome fusion, causing the accumulation of autophagosomes and, consequently, of both p62 and LC3-II.

Collectively, data from these experiments demonstrated that sorafenib-resistant HCC cell lines are chemosensitive to regorafenib, which has been shown to partially exert its anti-cancer actions by targeting the pro-survival FOXO3-induced autophagy. These findings confirm the implication of this novel mechanism in the refractoriness of HCC cells to sorafenib, indicating that its therapeutic inhibition could be beneficial to enhance the sorafenib response rate.

## 3. Discussion

Autophagy has been widely associated with the modulation of chemotherapy efficacy in cancer. However, this intracellular and self-digestive mechanism can either drive drug resistance or sensitivity, showing a double-edged role [32]. Contradictory roles of autophagy have been even reported in HCC cells treated with different chemotherapeutic agents, denoting the double function and context-dependency of autophagy. Activation of lethal autophagy has been shown to increment the cytotoxic effects of doxorubicin [33], cisplatin [34], and 5-fluorouracil [35] in several in vitro or in vivo HCC models. Nonetheless, other HCC investigations detected that autophagy induction enhances resistance to oxaliplatin [36] and epirubicin [37], among others.

Although sorafenib resistance has been related to autophagy, the exact implication of this dual process in sorafenib resistance acquisition remains largely unknown. In order to address this unmet need, we started with the characterization of the basal autophagic rate of our sensitive and sorafenib-resistant HCC in vitro models. HepG2S1 and HepG2S3 sorafenib-resistant lines exhibited induced basal autophagy compared to the non-resistant parental HepG2. In agreement with our findings, autophagy has shown to be induced in Hep3B-derived sorafenib-resistant cells [38,39], which was also confirmed in a xenograft tumor model [38]; as well as by independent works using sorafenib-refractory HepG2 [40], Huh7 [39,40,41,42] or derived in vivo models [42]. Therefore, there is predominant evidence supporting the relationship between sorafenib treatment and autophagy induction in HCC.

Functional autophagy has been traditionally linked to lower levels of p62 [24,25]. Here, we observed greater protein levels of p62 in the sorafenib-resistant cells, in which autophagy seems to be induced. However, an investigation carried out by Pan et al. [43] indicated that upregulation of p62 correlates with lesser sorafenib sensitivity in HepG2 cells, being also linked to a worse HCC prognosis [44]. It is known that p62 is highly controlled at the transcriptional level and that oxidative stress, such as chemotherapy administration produces, is able to stabilize the transcription factor NRF2 and thereby promote p62 transcription [26,27]. Additionally, NRF2 overexpression has been related to poor prognosis in solid malignancies [45]. In the present study, sorafenib-resistant cells displayed increased ROS levels, greater NRF2 protein expression and p62 mRNA levels. These results suggest that prolonged sorafenib treatment most likely enhanced the NRF2/p62 pathway. Consequently, p62 de novo synthesis appears to counteract p62 autophagic turnover in our chemoresistant cell lines, potentially conferring survival advantages.

Bafilomycin A1 and chloroquine are two specific inhibitors of autophagolysosome formation that are commonly used to block autophagic flux at late stages [24]. In our sorafenib-resistant lines, these compounds decreased autophagolysosome content but increased p62 and LC3-II protein accumulation. These are distinctive hallmarks of the suppression of a previously induced autophagy, demonstrating that our resistant cells are autophagy-competent and that sustained sorafenib administration triggered autophagy in the chemoresistant lines.

Although activation of autophagy is frequently associated with chemoresistance, some articles attributed an anti-tumor effect to autophagy during sorafenib resistance. Thus, research has reported that miR-21 contributes to sorafenib resistance by inhibiting a pro-death autophagy in both HepG2 and Huh7 sorafenib-resistant in vitro models [46]. Furthermore, Zhai et al. [47] proved that chronic exposition to sorafenib switches autophagy from a protective to a death-promoting role in HepG2 and Huh7 cells undergoing sorafenib resistance; something that was also seen under treatment with a phosphatidylinositol-4,5-bisphosphate 3-kinase (PI3K) and mammalian target of rapamycin (mTOR) inhibitor [41]. Otherwise, abolition of autophagy in vitro or in vivo has been shown to attenuate drug resistance to oxaliplatin [36], doxorubicin [48], or cisplatin [49].

In the current research, suppression of the overactivated autophagy by bafilomycin A1 or chloroquine increased sorafenib-resistant cell death. Therefore, we can conclude that instead of an anti-tumor role, autophagy is playing a cytoprotective action in our sorafenib-resistant HCC in vitro models that permits sorafenib-mediated cell death evasion. These findings are also supported by a study where administration of hydroxychloroquine modulates autophagy and oxidative stress to surpass sorafenib resistance in Huh7 cells displaying a resistant phenotype [50]. Similarly, chloroquine promoted the anti-tumor effect of sorafenib in vitro and in vivo [51]; while a combination of sorafenib plus 3-methyladenine, an inhibitor of autophagy at early phases, also enhanced the apoptotic rate in HCC [52]. Liang et al. [22] and Lin et al. [21] also showed that autophagy inhibition using 3-methyladenine promotes sorafenib actions under a specific hypoxic microenvironment. However, results from experiments using 3-methyladenine should be considered with caution and confirmed by other autophagy inhibitors, as this compound can exert opposed actions depending on the circumstances and is able to inhibit additional targets [24].

To elucidate the underlying mechanisms of autophagy-mediated sorafenib resistance has become an urgent health need, as well as the development of targeted therapies that satisfy the therapeutic requirements of advanced HCC patients. In this study, we focused on accurately determining the controversial and novel implication of FOXO3 in the autophagy-related sorafenib resistance acquisition, as well as on elucidating its potential modulation by the second-line drug regorafenib.

The two previous studies addressing this topic [21,22] explicitly reported opposed conclusions regarding FOXO3 and the modulation exerted on autophagy in sorafenib-refractory HCC. Specifically, Liang et al. [22] observed that hypoxia induces transcriptional activity of FOXO3, which subsequently promotes autophagy and reduces sorafenib sensitivity in HCC. Meanwhile, Lin et al. [21] suggested that FOXO3 is downregulated in sorafenib-resistant HCC under hypoxia, leading to autophagy activation and sorafenib resistance enhancement. Therefore, while one study proposes FOXO3 downregulation to inhibit autophagy and chemosensitize HCC cells under hypoxia [22], the other one contrarily indicates that FOXO3 overexpression could overcome sorafenib resistance due to autophagy suppression [21]. Moreover, it must be mentioned that Liang et al. [22] did not employ any sorafenib-resistant HCC in vitro or in vivo model throughout the whole investigation, using traditional sorafenib-sensitive HCC cell lines (Huh7, LM-3, SNU-387, and SNU-449), and an LM-3-derived xenograft model. This constitutes a significant experimental inaccuracy when studying the chemoresistance phenomenon, because these HCC cells still preserve sorafenib sensitivity and do not actually resemble chemoresistance conditions.

Here, we first analyzed FOXO3 expression in HCC samples and its involvement in patient survival, observing that FOXO3 levels in HCC tissues are significantly higher than those of healthy liver samples and finding that enhanced FOXO3 expression correlates with poor patient prognosis. Consistently, the investigations carried out by Ahn et al. [53] and Song et al. [54] reported that FOXO3 overexpression determines more aggressive HCC phenotypes.

Furthermore, we found that expression of multiple key autophagy-related genes is positively correlated with FOXO3 in HCC patients, suggesting that elevated FOXO3 levels could be related to possible protective autophagy induction in this cancer type. Given that FOXO3 was associated with more malignant HCC phenotypes and with a pro-autophagic microenvironment, we next addressed the hypothesis by which FOXO3 could mediate, at least in part, the activation of pro-survival autophagy in HCC cells during the acquisition of sorafenib resistance. As suspected, protein levels and nuclear translocation of FOXO3 in sorafenib-resistant cells were significantly higher, suggesting that FOXO3 expression and transcriptional activity are upregulated. Similarly, Zhou et al. [18] and Liu et al. [19] demonstrated that FOXO3 upregulation mediates the loss of sensitivity to doxorubicin, epirubicin, or cisplatin in HCC in vitro models. Likewise, the enhanced transcriptional activity of FOXO3 was found in oxaliplatin-resistant HCC cells [55].

Liang et al. [22] reported that hypoxia promotes nuclear retention of FOXO3 in traditional non-resistant HCC cell lines and is ultimately linked to the loss of sorafenib sensitivity under oxygen deprivation. In our investigation, we also observed FOXO3 nuclear translocation in HepG2S1 and HepG2S3 cells. However, it should be considered that our study was carried out under normal oxygen conditions and employed sorafenib-resistant in vitro models. Therefore, these findings should not be strictly compared.

We subsequently evaluated the involvement of this factor in the basal cytoprotective autophagy of HepG2S1 and HepG2S3. We transiently silenced FOXO3 and then we determined its direct impact on autophagy flux and survival rate of sorafenib-resistant cells. FOXO3 knockdown decreased its nuclear localization, suggesting an impaired transcriptional activity. Accordingly, FOXO3 downregulation reduced protein levels of its autophagy targets ULK1, Beclin-1 and LC3, and the autophagolysosome amount. Furthermore, silenced sorafenib-resistant lines showed decreased cell proliferation and viability, as well as enhanced pro-apoptotic markers expression. Collectively, these results indicate that FOXO3 upregulation plays a pro-survival role during the acquisition of sorafenib resistance in HCC by mediating the overactivation of protective autophagy. Meanwhile, the investigation by Liang et al. [22] denoted that the hypoxic microenvironment triggers FOXO3 activation, leading to autophagy induction and chemoresistance promotion. Nevertheless, this work was conducted with conventional sorafenib-treated but non-resistant Huh7, LM-3, SNU-387, and SNU-449 lines, additionally employing an LM-3 subcutaneous xenograft nude mice model. Therefore, both in vitro and in vivo models did not actually resemble chemoresistance conditions and results should be compared with caution [22]. Contrariwise, Lin et al. [21] suggested that FOXO3 overexpression could ameliorate n6-methyladenosine-related sorafenib sensitivity in HCC by autophagy inhibition under hypoxia.

In line with our findings, miR-223 has also been shown to target FOXO3-induced autophagy and rescue doxorubicin sensitivity in HCC [18], while osteopontin elicited epirubicin and cisplatin resistance through the upregulation of FOXO3-dependent autophagy [19]. Similar reports are found in neuronal [17] and cervical cancer cells [20].

Regorafenib, a sorafenib-like TKI with enhanced pharmacological activity, constitutes one of the main second-line treatments used on the HCC clinical setting when sorafenib fails [56]. The pre-clinical efficacy of this agent on chemosensitive HCC in vitro and in vivo models has been already proved [31,57]; finding that regorafenib could even palliate the loss of sorafenib sensitivity mediated by hepatocyte growth factor in the conventional non-resistant HCC cell lines SMMC-7721 and HepG2 [58]. Besides, it has been briefly suggested that autophagy modulation by regorafenib could account for its anti-tumor activity [31], but the potential regulatory effects of this multi-kinase inhibitor on FOXO3 and autophagy in sorafenib-resistant HCC has not been investigated yet. Thus, as a novelty, we finally decided to employ regorafenib to determine whether the efficacy of this second-line drug is also due to FOXO3 targeting and consequent inhibition of the cytoprotective autophagy in our sorafenib-resistant cells. If affirmative, this finding would help confirm that FOXO3-dependent induction of autophagy definitely contributes to sorafenib resistance acquisition in HCC.

Apart from ascertaining the efficacy of regorafenib to reduce sorafenib-resistant cell proliferation and viability, we proved for the first time that regorafenib causes FOXO3 downregulation and autophagic flux blockage in sorafenib-resistant HCC. These novel results indicate that regorafenib targets such FOXO3-mediated autophagy, promoting sorafenib-resistant cell death. Curiously, regorafenib increased both p62 and LC3-II expression, suggesting that regorafenib could also cause autophagy inhibition at the last stages. To analyze this possibility, we employed bafilomycin A1 in single or combined treatment with regorafenib and compared the impact on protein levels. Individual or conjunct administration enhanced p62 and LC3-II levels, but co-treatment did not further accumulate them compared to specific single treatments. These novel findings indicate that regorafenib can also impair autophagy at late phases, blocking autophagosome-lysosome fusion. Contrariwise, preceding HCC evidence reported that regorafenib could induce autophagy in conventional sorafenib-sensitive HCC cells [31]. Here, we used adequate sorafenib-resistant in vitro models; thus, previous findings cannot be actually compared with the results displayed in our investigation. We have to remember that regorafenib is a second-line drug approved for sorafenib-refractory HCC. Hence, the most adequate approach to investigate regorafenib effects at the pre-clinical level is to employ well-established sorafenib-resistant models such as those in the present work. Otherwise, using traditional HCC cells with this aim would show regorafenib efficacy as a first-line treatment.

Altogether, these results disclosed for the first time that the anti-tumor activity of regorafenib as a second-line drug for advanced HCC also relies on the FOXO3-induced cytoprotective autophagy inhibition, thereby supporting the involvement of this pro-survival mechanism in the sorafenib resistance acquisition.

## 4. Materials and Methods

### 4.1. Cell Culture and Reagents

Human HCC HepG2 cells were obtained from the American Type Culture Collection (Manassas, VA, USA) and two well-established sorafenib-resistant HepG2-derived cell lines (HepG2S1 and HepG2S3) were independently generated by the Laboratory of Hepatology of the University Hospitals Leuven, as described by van Malenstein et al. [59]. Cells were grown in Dulbecco’s Modified Eagle’s Medium (DMEM)-high glucose (Sigma-Aldrich, San Luis, MO, USA), supplemented with 10% fetal bovine serum and penicillin/streptomycin (100 U/mL) (Gibco™, Gaithersburg, MD, USA). Besides, they were cultured under a humidified 5% CO_2_ atmosphere at 37 °C. Cell media of sorafenib-resistant HCC cell lines always contained 6 μM sorafenib (Santa Cruz Biotechnology, Dallas, TX, USA) to maintain the chemoresistance.

Autophagic flux was modulated using bafilomycin A1 (Tocris Bioscience, Bristol, UK) and chloroquine (Sigma-Aldrich), two autophagy inhibitors, or rapamycin (MedChemExpress, Monmouth Junction, NJ, USA), an autophagy inductor. Regorafenib (Selleckchem, Houston, TX, USA), a second-line drug approved for sorafenib-refractory HCC [31], was also employed.

### 4.2. Acridine Orange Staining

Cells seeded in 8-chamber culture slides were washed with phosphate-buffered saline (PBS) and incubated for 15 min at 37 °C with 1 μg/mL acridine orange (Sigma-Aldrich). Then, the excess dye was removed by washing with PBS. Samples were air-dried, mounted, and briefly visualized in the Nikon Eclipse E600 microscope (Nikon Instruments Inc., Melville, NY, USA). The images were analyzed with NIS-Elements (Nikon Instruments Inc.) and ImageJ (National Institutes of Health, Bethesda, MD, USA) software, employing the CTCF formula and calculating the red/green CTCF ratio [60].

### 4.3. Immunofluorescence and Laser Confocal Imaging

Cells were seeded in 24-well plates containing coverslips coated with 0.2% gelatin from bovine skin (Sigma-Aldrich). Cells were washed with PBS and immediately fixed with 4% formaldehyde solution (Thermo Fisher Scientific, Waltham, MA, USA) for 15 min, permeabilized with 0.2% saponin (Sigma-Aldrich) for 20 min, and blocked with 1% fatty acid-free bovine serum albumin (Sigma-Aldrich) in PBS for 30 min. All steps were carried out at room temperature, washing three times with PBS between each of them. Cells were incubated overnight at 4 °C with primary antibodies against LC3 (1:500, PM036, MBL International, Woburn, MA, USA), LAMP2 (1:100, ab25631, Abcam, Cambridge, UK), FOXO3 (1:600, #99199, Cell Signaling Technology, Danvers, MA, USA) or Ki67 (1:200, sc-23900, Santa Cruz Biotechnology). Then, cells were washed with PBS and incubated for 1 h at room temperature with the following secondary antibodies: anti-rabbit conjugated with Alexa Fluor^®^ 647 (1:1000, ab150079, Abcam) or anti-mouse conjugated with Alexa Fluor^®^488 (1:1000, ab150113, Abcam). The coverslips were washed with PBS and mounted on glass slides with the mounting medium Fluoroshield^TM^ (Sigma-Aldrich) containing 4′6-diamidino-2-phenylindole (DAPI) for nuclei staining. Results were visualized in a Zeiss LSM 800 confocal laser scanning microscope (Zeiss, Jena, Germany). The images were analyzed using ZEN (Zeiss) and ImageJ software, employing the CTCF formula or the colocalization colormap plugin. For each immunofluorescence plus confocal microscopy assay (including colocalization experiments), images containing a similar number of cells within the same experimental condition were selected. As shown by immunocytochemistry images displayed in our Figures, the exact number of cells/image slightly varied depending on the cell line or experiment performed. In order to address this variability source, we always relativized CTCF data by the number of cellular nuclei found in the corresponding selected image before performing the statistical analysis.

### 4.4. Western Blot

Cells were trypsinized and subsequently placed in a homogenization buffer containing 0.25 mM sucrose, 10 mM Tris, and 1 mM EDTA (pH 7.4), as well as protease and phosphatase inhibitor cocktails (Roche Diagnostics, Basel, Switzerland). Cells were lysed by sonication during two pulses of 20 s at 60% amplitude and centrifuged at 14 × 10^3^ rpm for 10 min. Equal amounts of protein were separated by sodium dodecyl sulfate-polyacrylamide gel electrophoresis (SDS-PAGE) and transferred to polyvinylidene difluoride (PVDF) membranes (Bio-Rad, Hercules, CA, USA). Membranes were blocked for 1 h at room temperature employing 5% milk powder in a PBS solution with Tween 20 (Sigma-Aldrich) at 0.05% (PBS-T) and incubated overnight at 4 °C with the primary antibodies indicated in Table S1. After washing three times with PBS-T, the membranes were incubated for 1 h at room temperature with anti-rabbit (1:20000, 31460, Thermo Fisher Scientific) or anti-mouse (1:5000, P0260, Agilent Dako, Santa Clara, CA, USA) horseradish peroxidase (HRP)-conjugated secondary antibodies. Proteins were visualized using Pierce™ ECL western blotting substrate (Thermo Fisher Scientific) and densitometry reading of each band was performed using ImageJ software. Full-length immunoblots are shown in Figures S2–S10.

LC3 primary antibody can potentially detect both LC3-I (~16 kDa) and LC3-II (lipidated, ~14 kDa) forms. However, LC3-I is difficult to be detected by primary antibodies, which have a stronger affinity for LC3-II. Therefore, instead of using the traditional LC3-II/LC3-I ratio (cytosolic/membrane-bound LC3) as an indicator of autophagy-related structures, we followed recent guidelines that recommend quantifying LC3-II vs. the housekeeping protein [24,25].

### 4.5. ROS Measurement

Cells were washed with PBS and incubated with 20 μM 2′,7′-dichlorofluorescin diacetate (Sigma-Aldrich) for 30 min at 37 °C. Fluorescence was rapidly measured with the Synergy™ HT Multi-Mode Microplate Reader (BioTek Instruments Inc., Winooski, VT, USA) plus Gen 5 software (BioTek Instruments Inc.) using 485 nm/528 nm as exciting/emission wavelengths. H_2_O_2_ was employed as a positive control.

### 4.6. Real-Time Reverse Transcription Polymerase Chain Reaction (qRT-PCR)

Total RNA was isolated using TRIzol^TM^ Reagent (Invitrogen, Waltham, MA, USA) and quantified using the Nanodrop™ ND-1000 Spectrophotometer (Thermo Fisher Scientific). Residual DNA was removed using RQ1 RNase-free DNase Kit (Promega, Madison, WI, USA) and, afterwards, total RNA was reverse transcribed to cDNA using the High-Capacity cDNA Reverse Transcription Kit (Applied Biosystems, Waltham, MA, USA). cDNA was amplified in the StepOnePlus™ Real-Time PCR System using TaqMan™ Gene Expression Master Mix and commercial probes for p62 (Hs01061912_m1) and 18S rRNA (Hs99999901_s1) (Applied Biosystems), which was used as endogenous control. All procedures were performed according to the manufacturer’s instructions. Relative changes in gene expression levels were detected by the 2^−ΔΔCt^ method.

### 4.7. Assessment of SubG1 Cell Population by Flow Cytometry

Cells were trypsinized and centrifuged at 350× *g* for 5 min at 4 °C, washed with ice-cold PBS, and centrifuged again at the same conditions. Then, cells were fixed with 70% ethanol in PBS for 2 h at 4 °C and, afterwards, around 1 × 10^6^ cells/sample were centrifuged at 850× *g* for 5 min and washed with PBS. After another centrifugation cycle under these conditions, each sample was incubated with 0.5 mL PI/RNase Staining Buffer (BD Pharmingen™, San Jose, CA, USA) for 15 min at room temperature. Finally, stained cells were diluted with PBS and maintained at 4 °C. Using CyAn™ ADP flow cytometer (Beckman Coulter, Brea, CA, USA) and the Summit software (Beckman Coulter), 5 × 10^3^ events per sample were acquired. The percentage of subG1 cells was determined using Flowing software (Turku Bioscience, Turku, Finland).

### 4.8. Cell Viability Assays

Cell viability was measured by 3-(4,5-dimethylthiazol-2-yl)-2,5-diphenyl-tetrazolium bromide (MTT) (Sigma-Aldrich) assay or by CellTiter-Glo^®^ Luminescent Cell Viability Assay (Promega). For MTT assay, cell media were removed and, after washing with PBS, cells were incubated for 3 h at 37 °C with a 1:10 serum-free medium solution containing 5 mg/mL MTT. Then, media were replaced by dimethyl sulfoxide (DMSO) to dissolve formazan crystals. Finally, absorbance at 570 nm was measured with the Synergy™ HT Multi-Mode Microplate Reader and Gen 5 software. On the other hand, the CellTiter-Glo^®^ assay was performed according to the manufacturer’s instructions. Luminescence was also measured with the Synergy™ HT Multi-Mode Microplate Reader and Gen 5 software.

### 4.9. Expression, Survival and Gene Correlation Analysis of FOXO3 in HCC Patient Samples

TCGA analysis tool from the UALCAN database, a comprehensive web resource for evaluating cancer OMICS [61] (http://ualcan.path.uab.edu/analysis.html, accessed on 26 April 2021), was used for the comparison of FOXO3 levels between normal liver and HCC samples, the comparative survival analysis between HCC patients with low/medium and high FOXO3 expression, and the determination of autophagy-related genes whose expression correlates with FOXO3 in HCC patients. Specifically, “FOXO3” gene was entered into the TCGA Gene analysis tool and “Liver hepatocellular carcinoma” was selected as the cancer type of interest. Then, “Expression”, “Survival” and “Correlation” were checked. For the evaluation of gene expression correlation, we searched for the potential positive or negative correlation between FOXO3 and genes involved in the autophagy KEGG pathway (hsa04140) encoding autophagic proteins with relevant roles on autophagosomes formation, maturation, fusion with lysosomes, and cargo degradation.

### 4.10. Gene Silencing

Cells were seeded in 6-well plates and, next day, ON-TARGETplus Human FOXO3 siRNA SMARTPool (a mixture of 4 siRNA targeting FOXO3) and ON-TARGETplus Non-targeting Control Pool (a negative control pool of 4 siRNA) were introduced into cells using the DharmaFECT 4 Transfection Reagent (Horizon Discovery, Waterbeach, UK) following the manufacturer’s protocol. Eight hours after transfection, media were replaced by supplemented DMEM-high glucose media and cells were reseeded according to the different experiments.

### 4.11. Crystal Violet Staining

Cells were washed with ice-cold PBS and fixed with 4% formaldehyde for 15 min. Afterwards, cells were washed with PBS and stained for 20 min with 0.1% crystal violet solution (Labkem, Barcelona, Spain) dissolved in 10% ethanol. After washing three times with Milli-Q water, 10% acetic acid was incorporated, maintaining under shaking for 20 min to dissolve the crystals. Absorbance was finally measured at 590 nm using the Synergy™ HT Multi-Mode Microplate Reader and Gen 5 software.

### 4.12. Statistical Analysis

The *p*-values from FOXO3 expression and survival evaluation in HCC patient samples, as well as the Pearson-CC values for gene correlation assessment, were provided by the UALCAN database. Meanwhile, significance for the correlation analysis in HCC samples and all other results (expressed as mean values ± SD) were analyzed by the statistical package GraphPad Prism 6 (GraphPad Software, San Diego, CA, USA). According to the different in vitro experiments, unpaired *t*-test, one-way or two-way ANOVA followed by Dunnett or Tukey post hoc tests were employed. Statistical significant was considered when *p* < 0.05.

## 5. Conclusions

In conclusion, this study evidenced that overactivation of pro-survival autophagy due to aberrant upregulation of FOXO3 plays a pivotal role in the acquisition of sorafenib resistance in HCC. Our work provides relevant information about the molecular mechanisms behind the complex process of development of chemoresistance, strongly contributing to unraveling the controversial role of FOXO3 on autophagy modulation in sorafenib-resistant HCC. This research reported that FOXO3 is related to enhanced autophagy and predicts poor prognosis and a more malignant HCC phenotype; thus, FOXO3 represents a promising biomarker. Furthermore, regorafenib, one of the main second-line drugs administered when sorafenib fails, demonstrated for the first time that its anti-tumor action in sorafenib-refractory HCC is mediated, at least in part, through FOXO3 downregulation and autophagy abolition. Therefore, molecular targeting of FOXO3 could be useful to prevent or overcome HCC sorafenib resistance by autophagy inhibition. Altogether, these results contribute to firmly establishing the interplay between FOXO3, autophagy and sorafenib resistance in this cancer type, supporting this new molecular mechanism that accounts for chemoresistance and opening a new therapeutic window to achieve better HCC patient outcomes.

## Figures and Tables

**Table 1:** Autophagy-related genes positively and significantly correlated with FOXO3 in human HCC samples.

Gene Symbol	Full Name	Pearson-CC
ATG5	Autophagy-related 5	+0.62
RAB33B	RAB33B, member RAS oncogene family	+0.6
STX17	Syntaxin 17	+0.56
ATG2B	Autophagy-related 2B	+0.55
SMCR8	SMCR8-C9orf72 complex subunit	+0.55
TRAF6	TNF receptor associated factor 6	+0.55
UVRAG	UV radiation resistance associated	+0.55
PIK3C3	Phosphatidylinositol 3-kinase catalytic subunit type 3	+0.51
PIK3R4	Phosphoinositide-3-kinase regulatory subunit 4	+0.51
TANK	TRAF family member associated NFKB activator	+0.51
ATG16L1	Autophagy-related 16 like 1	+0.48
HMGB1	High mobility group box 1	+0.48
AMBRA1	Autophagy and beclin-1 regulator 1	+0.47
TBK1	TANK binding kinase 1	+0.47
RAB1A	RAB1A, member RAS oncogene family	+0.44
RAB7A	RAB7A, member RAS oncogene family	+0.42
ULK1	Unc-51 like autophagy activating kinase 1	+0.41
SH3GLB1	SH3 domain containing GRB2 like, endophilin B1	+0.4
ATG16L2	Autophagy-related 16 like 2	+0.38
ATG4C	Autophagy-related 4C cysteine peptidase	+0.38
ATG9A	Autophagy-related 9A	+0.38
ATG12	Autophagy-related 12	+0.37
NRBF2	Nuclear receptor binding factor 2	+0.37
SNAP29	Synaptosome associated protein 29	+0.37
C9orf72	C9orf72-SMCR8 complex subunit	+0.36
DAPK1	Death associated protein kinase 1	+0.36
ATG3	Autophagy-related 3	+0.35
WDR41	WD repeat domain 41	+0.35
RB1CC1	RB1 inducible coiled-coil 1	+0.34
BECN1	Beclin-1	+0.31
ATG4B	Autophagy-related 4B cysteine peptidase	+0.3

Pearson-CC, Pearson-correlation coefficient.

## Data Availability

Publicly available datasets were analyzed in this study. This data can be found here: http://ualcan.path.uab.edu/analysis.html (accessed on 26 April 2021).

## Supplementary Materials

The following are available online at https://www.mdpi.com/article/10.3390/ijms222111770/s1.
