# Supplementary material for: Autophagy-Related Chemoprotection against Sorafenib in Human Hepatocarcinoma: Role of FOXO3 Upregulation and Modulation by Regorafenib
Source: Int J Mol Sci. 2021 Oct 29;22(21):11770. doi: 10.3390/ijms222111770 (PMC8583804; doi:10.3390/ijms222111770)
Supplement: Supplementary file 1 [file ijms-22-11770-s001.zip › ijms-1399277 - sm.pdf]

International Journal of *Molecular Sciences*

MDPI

Supplementary Materials

Autophagy-Related Chemoprotection against Sorafenib in Human Hepatocarcinoma: Role of FOXO3 Upregulation and Modulation by Regorafenib

Flavia Fondevila [1,2], Carolina Méndez-Blanco [1,2], Paula Fernández-Palanca [1,2], Tania Payo-Serafín [1,2], Jos van Pelt [3], Chris Verslype [3], Javier González-Gallego [1,2,†] and José L. Mauriz [1,2,*,†]

1 Campus de Vegazana s/n, University of León, Institute of Biomedicine (IBIOMED), 24071 León, Spain; ffonp@unileon.es (F.F.); cmenb@unileon.es (C.M.-B.); pferp@unileon.es (P.F.-P.); tpayos00@estudiantes.unileon.es (T.P.-S.); jgonga@unileon.es (J.G.-G.)

2 Centro de Investigación Biomédica en Red de Enfermedades Hepáticas y Digestivas (CIBERehd),
Instituto de Salud Carlos III, Av. de Monforte de Lemos 5, 28029 Madrid, Spain

3 Laboratory of Clinical Digestive Oncology, Department of Oncology, Leuven Cancer Institute (LKI), KU Leuven and University Hospitals Leuven, Leuven, Belgium; jos.vanpelt@kuleuven.be (J.v.P.); chris.verslype@uzleuven.be (C.V.)

* Correspondence: jl.mauriz@unileon.es; Tel.: +34 987291981

† J.L.M. and J.G.-G. share senior authorship.

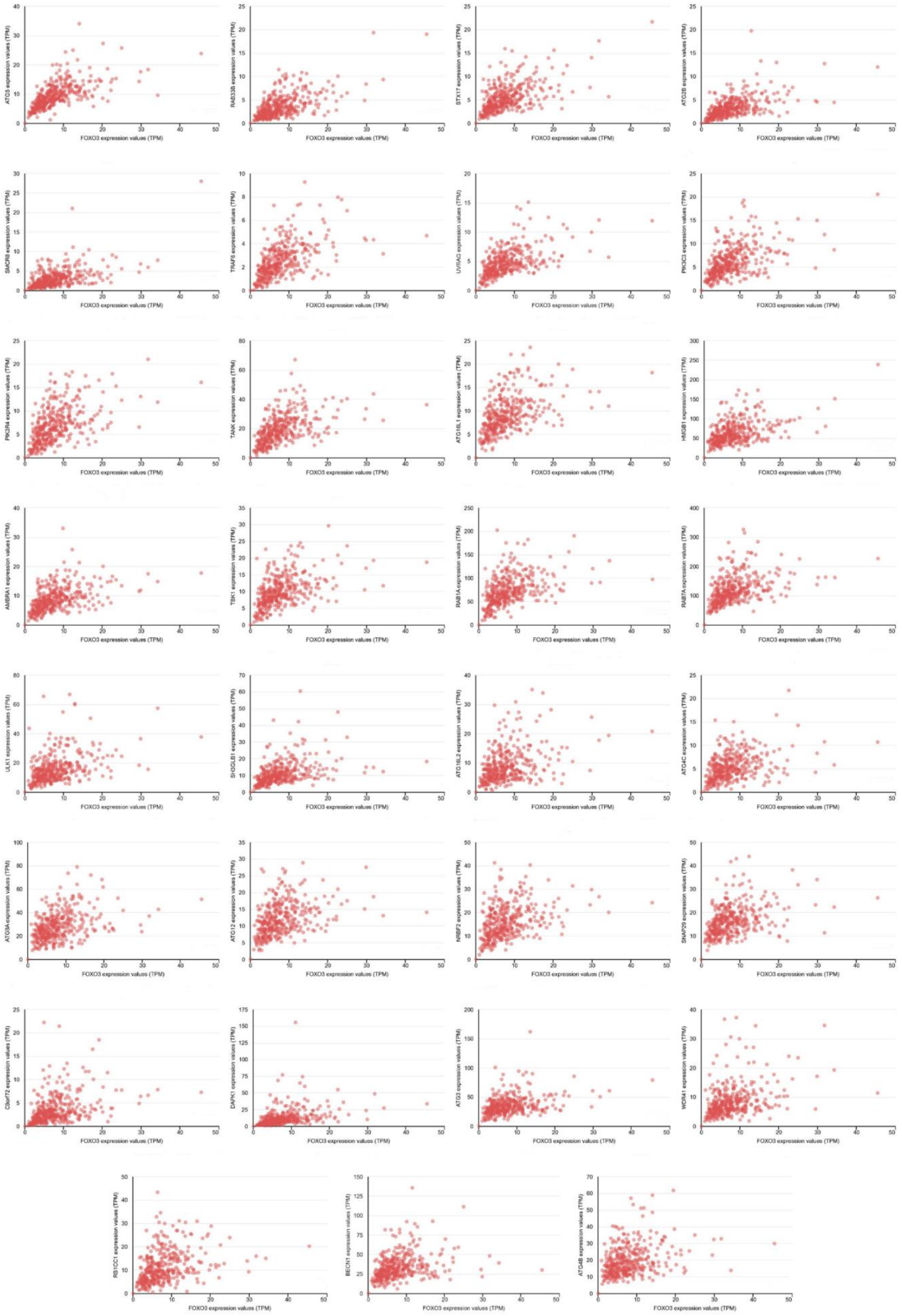

Figure S1. Gene expression correlation plots for a subset of autophagy-related genes involved in the autophagy KEGG pathway (hsa04140) that are positively and significantly correlated with FOXO3 expression in human HCC samples. TPM, transcripts per million.

Table S1. Primary antibodies employed for protein detection by Western blot.

Protein	Reference	Source	Antibody Host and Type	Molecular Weight (kDa)	Dilution
Atg5	#12994	Cell Signaling Technology	Rabbit monoclonal	55	1:1000
Bax	sc-493	Santa Cruz Biotechnology	Rabbit polyclonal	23	1:200
Beclin-1	sc-11427	Santa Cruz Biotechnology	Rabbit polyclonal	60	1:200
Cleaved caspase-3	#9661	Cell Signaling Technology	Rabbit polyclonal	17/19	1:1000
FOXO3	#99199	Cell Signaling Technology	Mouse monoclonal	82–97	1:200
LC3	PM036	MBL International	Rabbit polyclonal	14/16	1:1000
NRF2	sc-722	Santa Cruz Biotechnology	Rabbit polyclonal	61–68	1:200
p62	#5114	Cell Signaling Technology	Rabbit polyclonal	62	1:1000
PCNA	sc-56	Santa Cruz Biotechnology	Mouse monoclonal	36	1:200
ULK1	#8054	Cell Signaling Technology	Rabbit monoclonal	150	1:1000
UVRAG	#5320	Cell Signaling Technology	Rabbit polyclonal	90	1:500

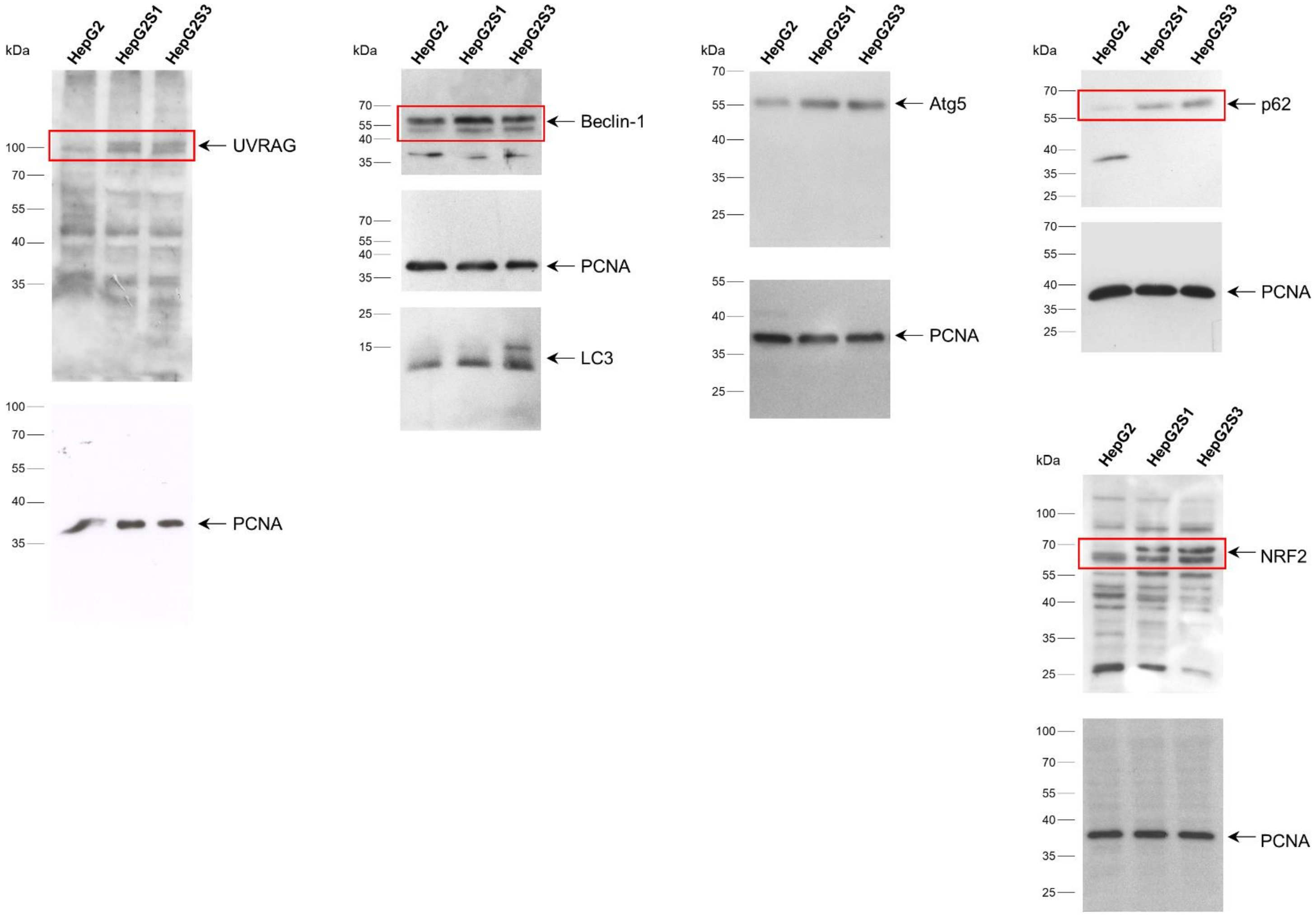

Figure S2. Full-length immunoblots from Figure 1.

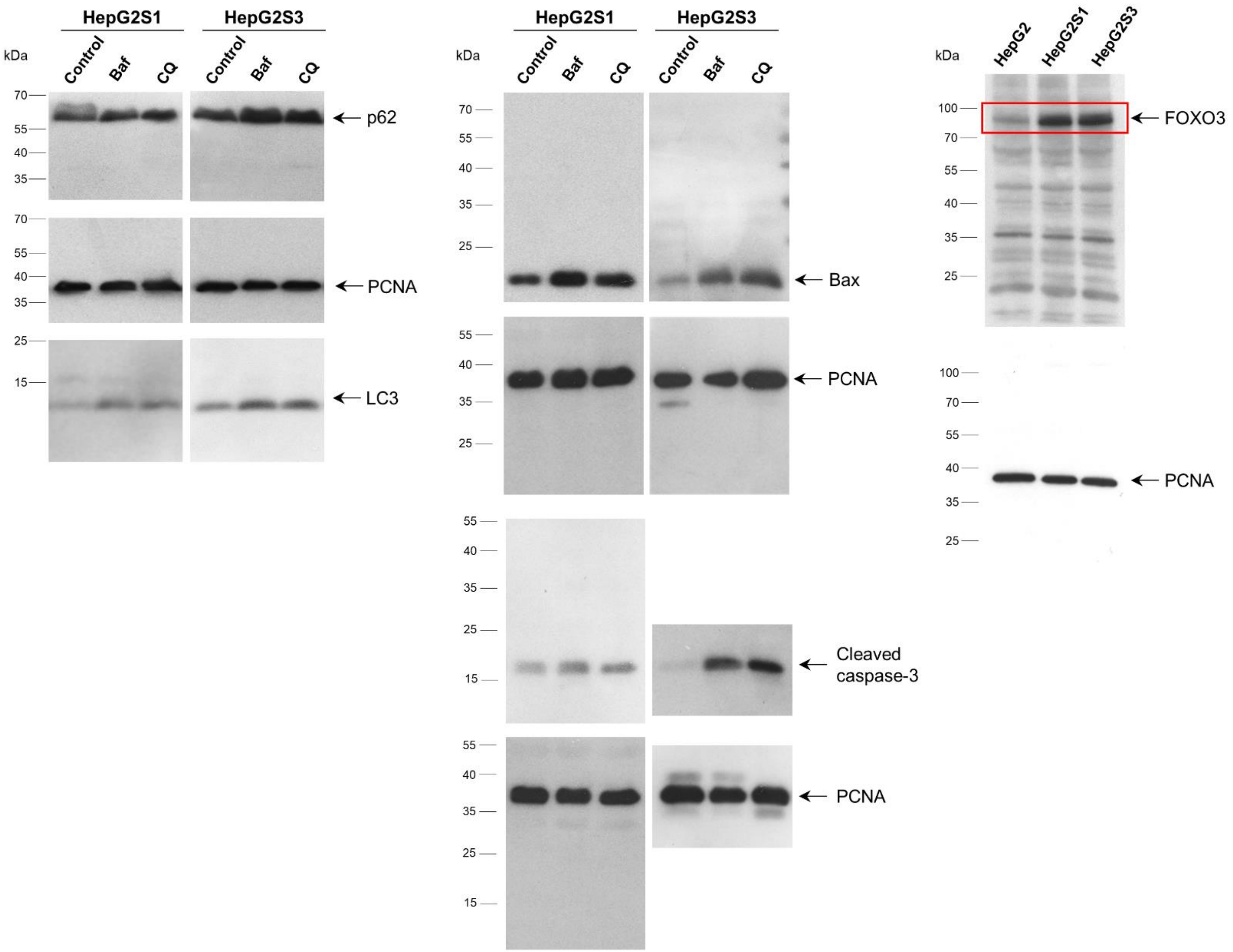

Figure S3. Full-length immunoblots from Figures 2 and 3.

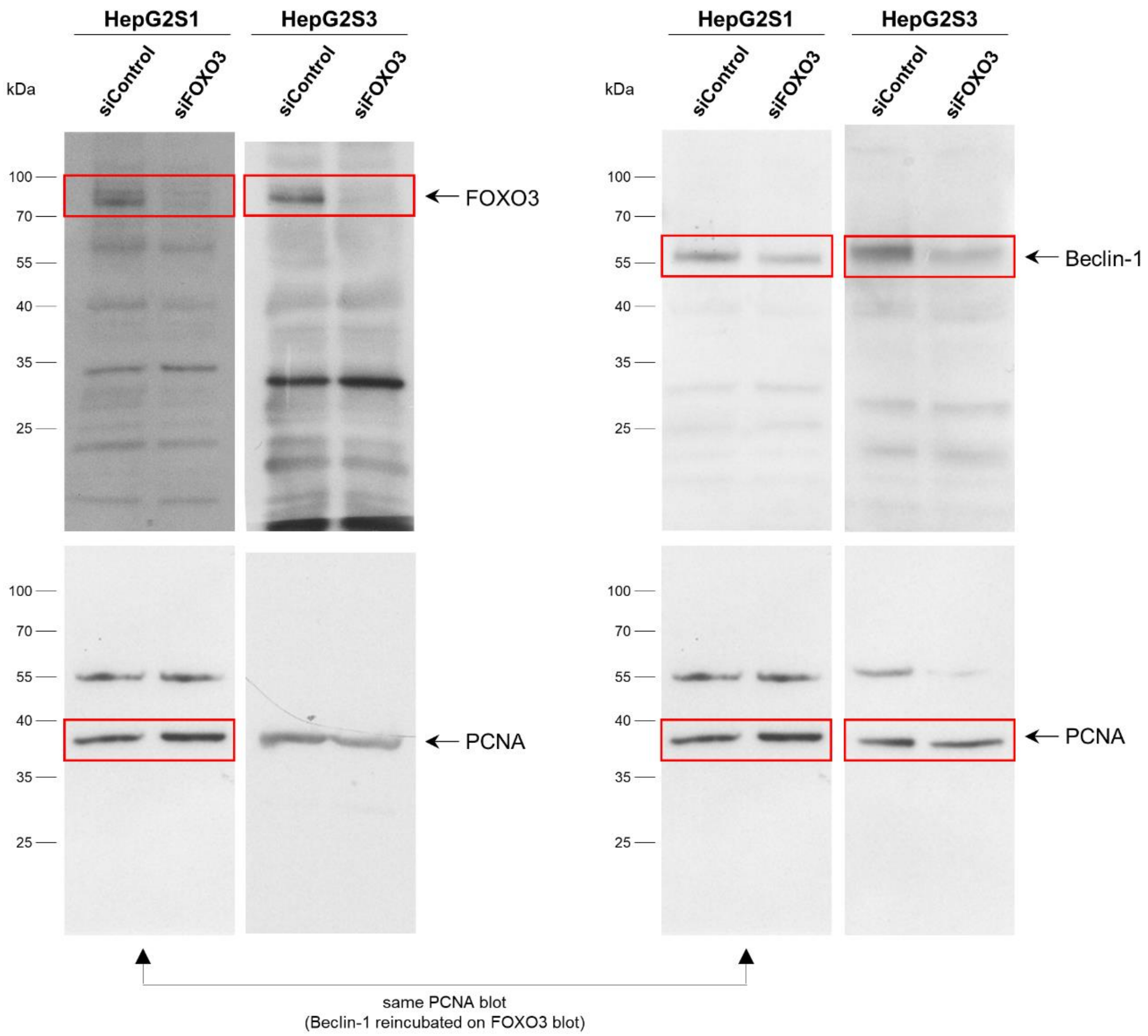

Figure S4. Full-length immunoblots from Figure 4 (FOXO3, Beclin-1 and corresponding PCNA).

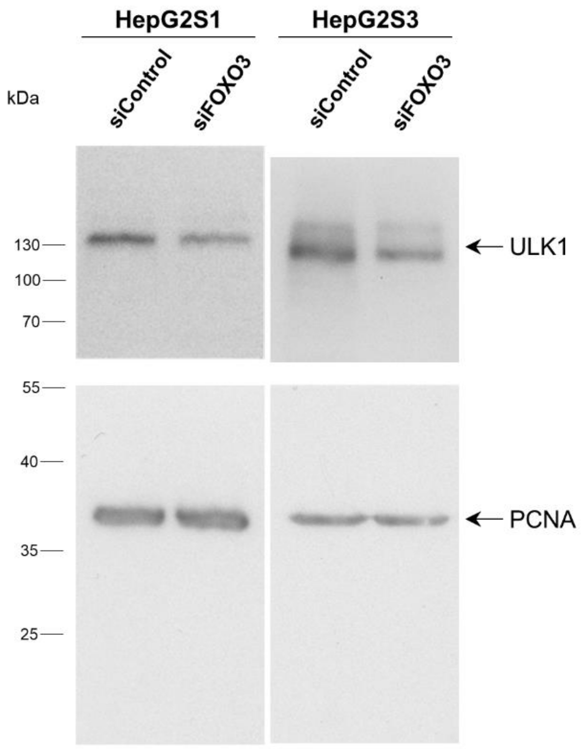

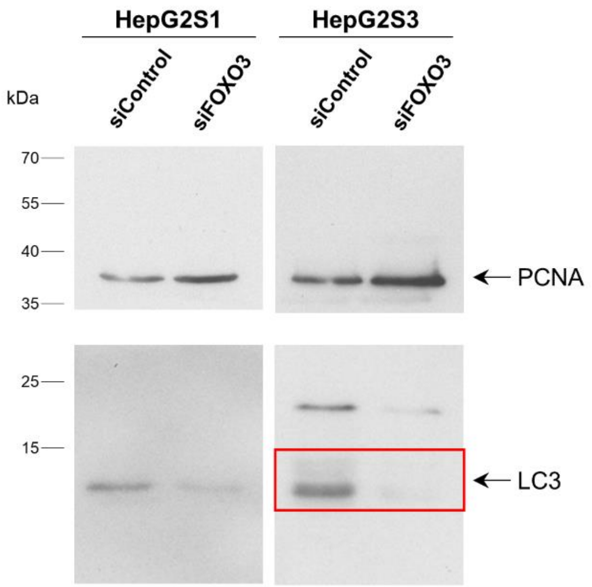

Figure S5. Full-length immunoblots from Figure 4 (ULK1, LC3 and corresponding PCNA).

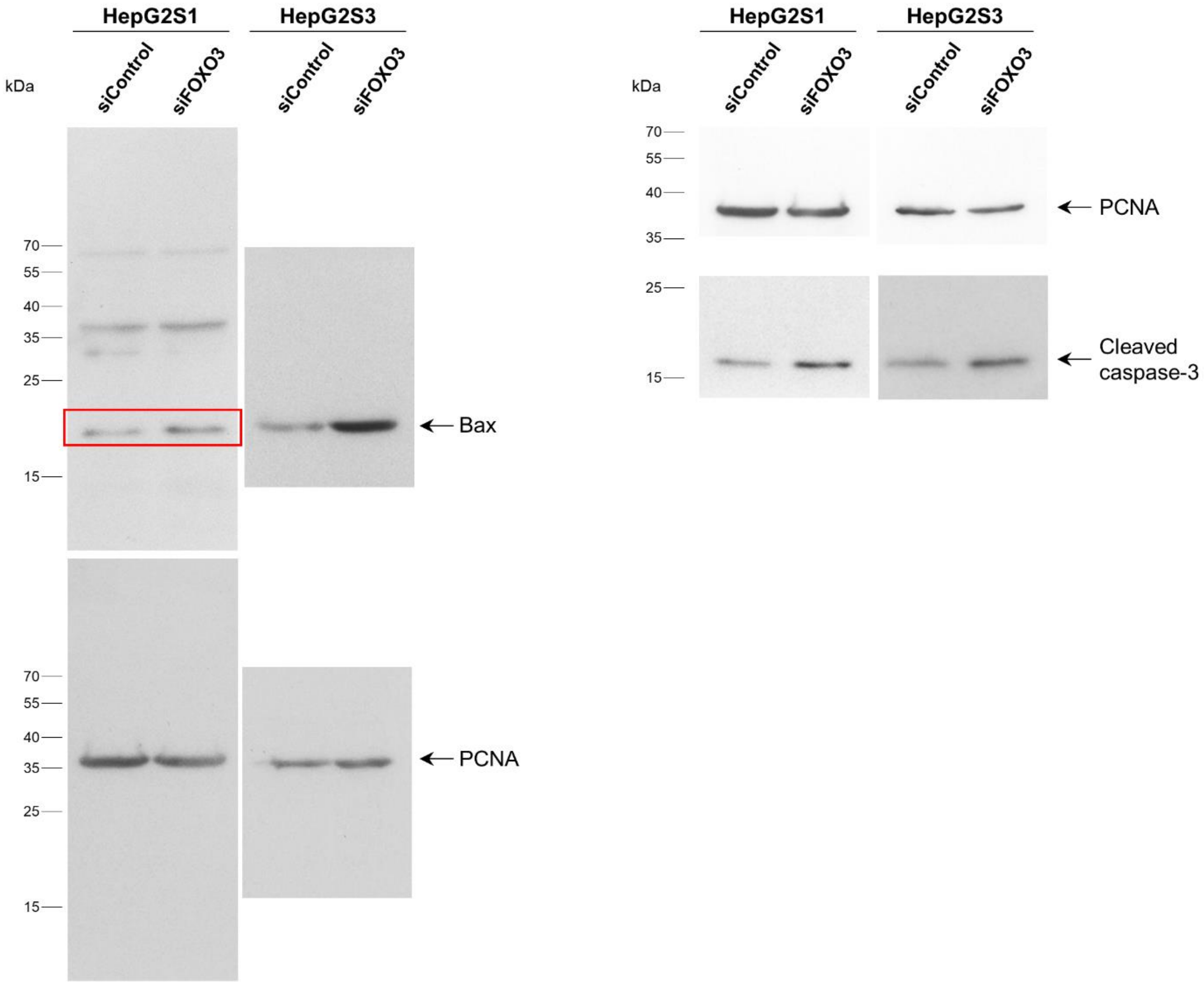

Figure S6. Full-length immunoblots from Figure 5.

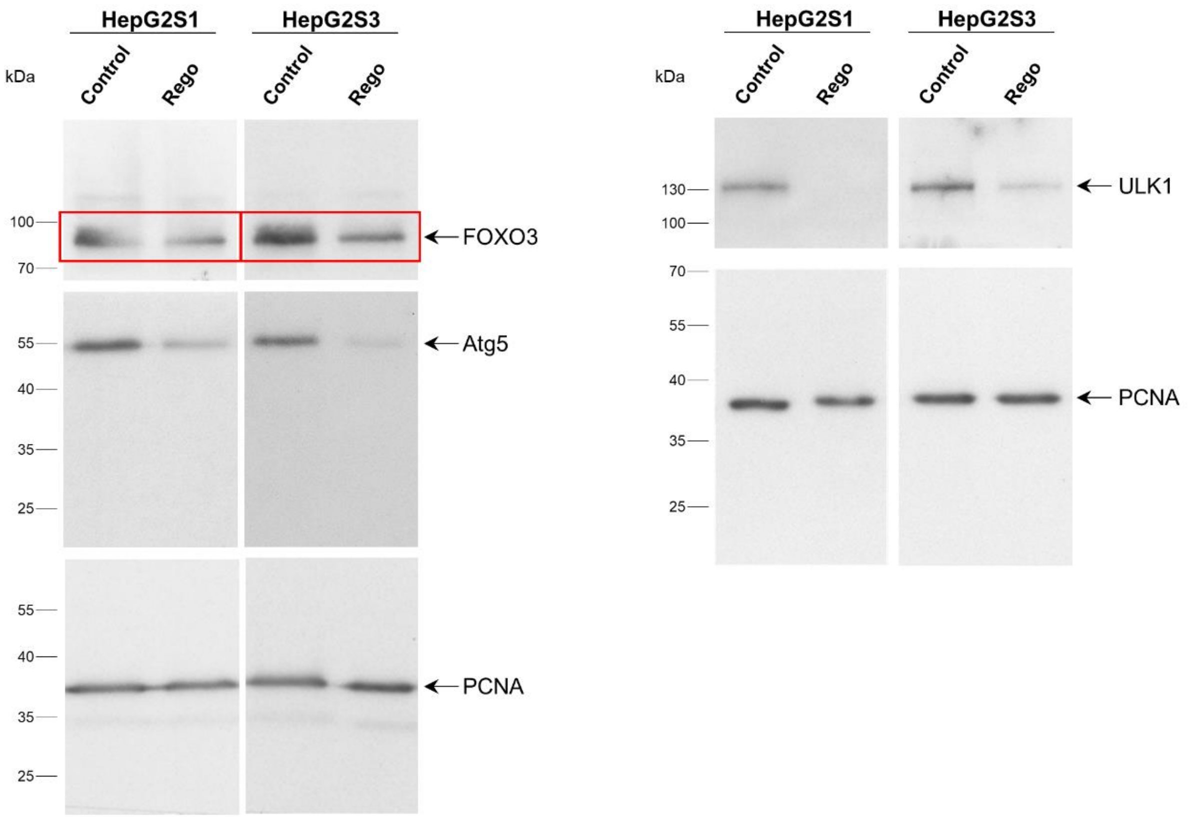

Figure S7. Full-length immunoblots from Figure 7a (FOXO3, Atg5, ULK1 and corresponding PCNA).

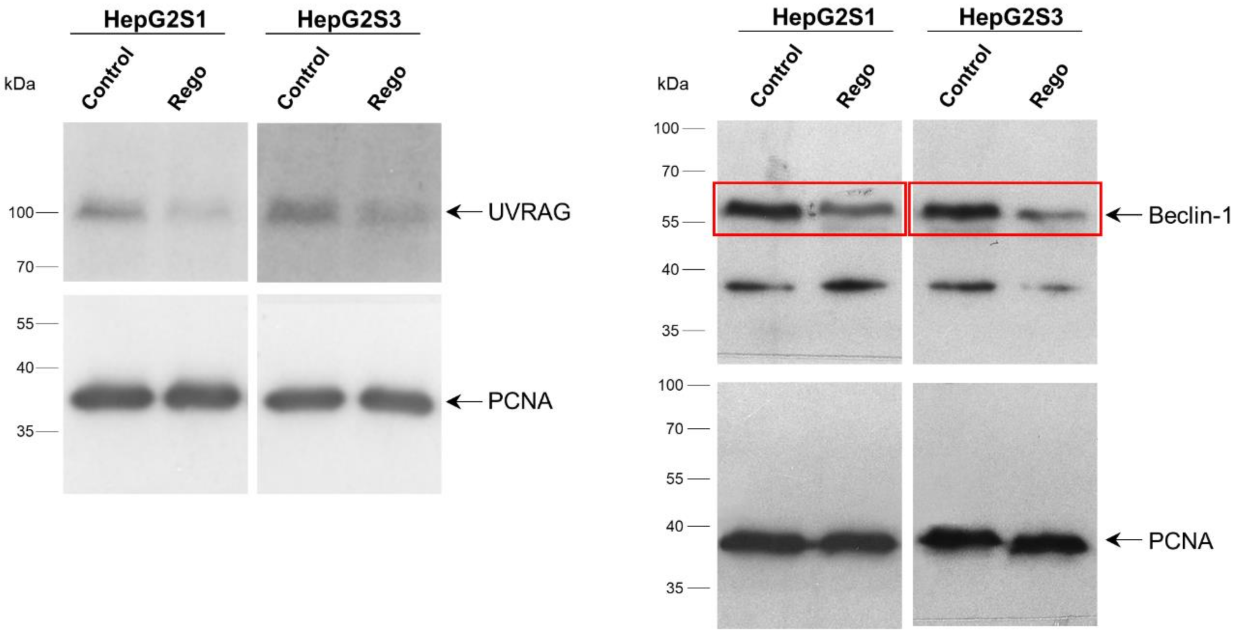

Figure S8. Full-length immunoblots from Figure 7a (UVRAG, Beclin-1 and corresponding PCNA).

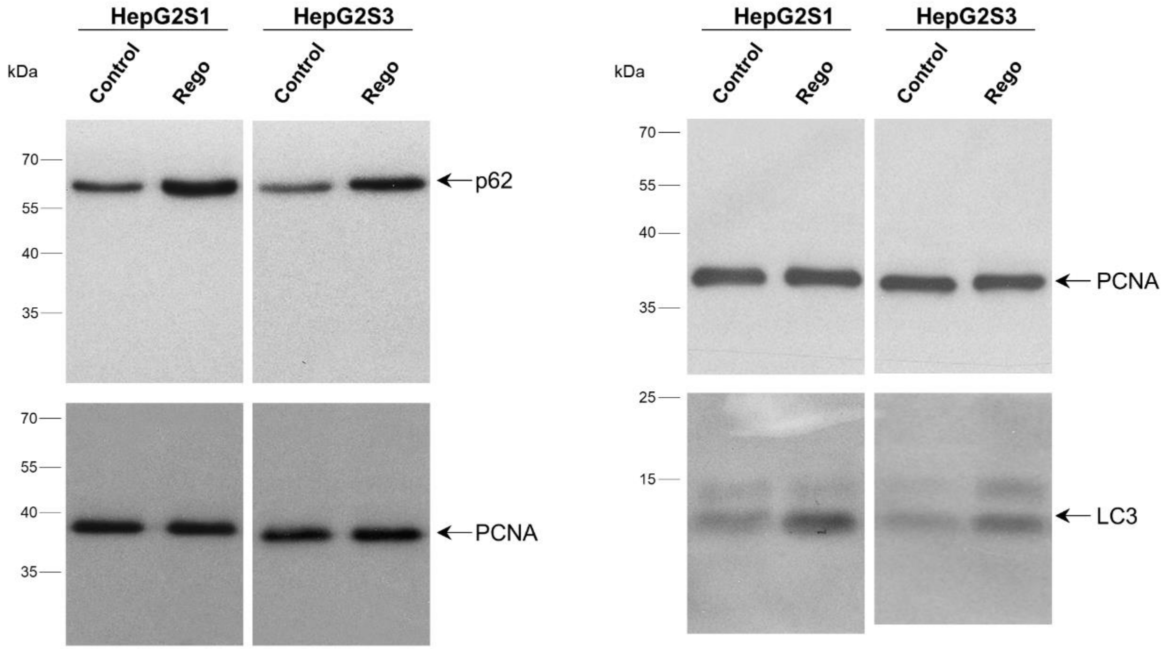

Figure S9. Full-length immunoblots from Figure 7a (p62, LC3 and corresponding PCNA).

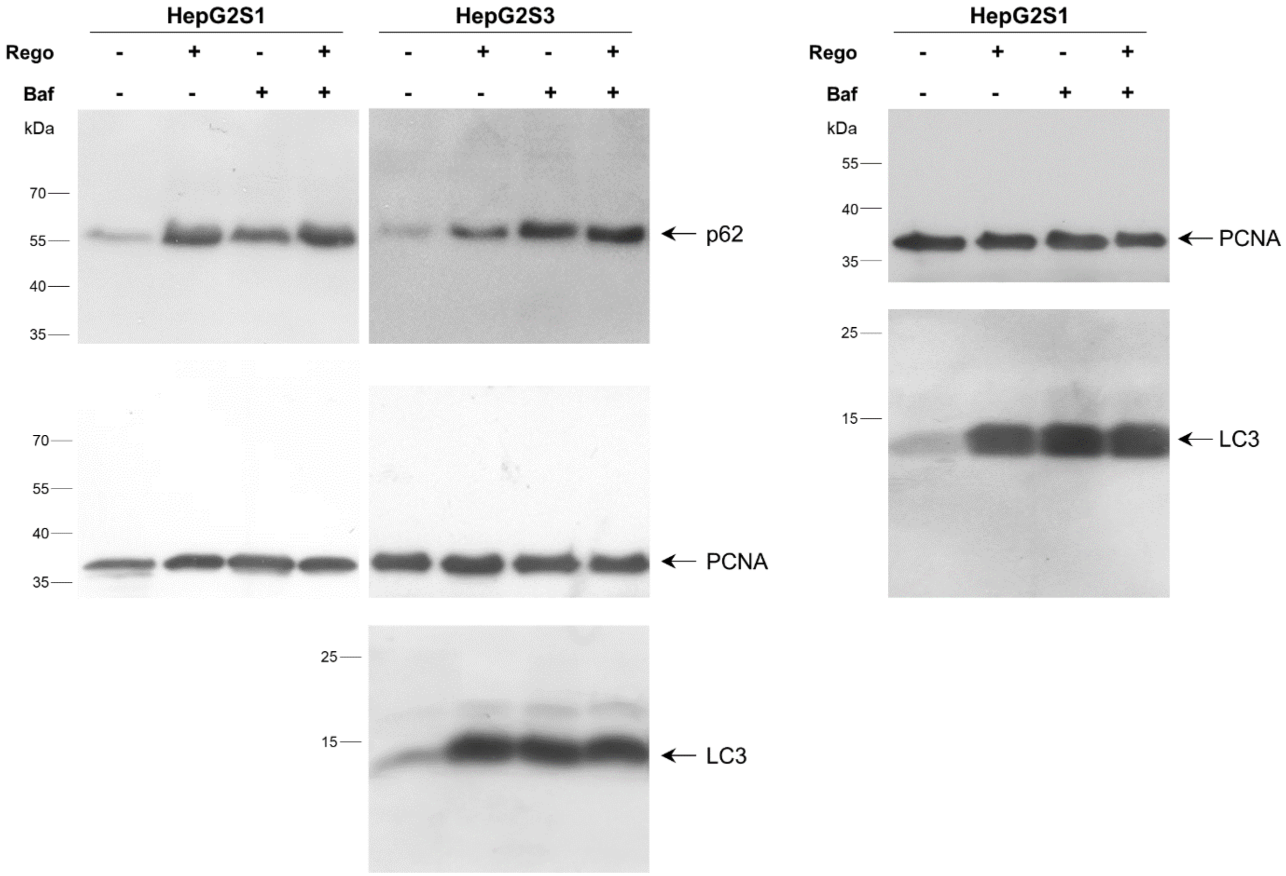

Figure S10. Full-length immunoblots from Figure 7e.
